# Suppressing Surface Degradation in Na‐Rich Prussian Blue Cathodes via Liquid‐Phase Dehydration

**DOI:** 10.1002/adma.73507

**Published:** 2026-05-27

**Authors:** Seunghye Jang, Hyebin Jeong, Jun Choi, Jeongsoo Hong, Jooyoung Jang, Jongyoon Han, Juwon Kim, Sang‐Min Lee, Changshin Jo

**Affiliations:** ^1^ Department of Battery Engineering Graduate Institute of Ferrous & Eco Materials Technology Pohang University of Science and Technology (POSTECH) Pohang Gyeongbuk Republic of Korea; ^2^ Department of Chemical Engineering Pohang University of Science and Technology (POSTECH) Pohang Gyeongbuk Republic of Korea; ^3^ Research Institute of Industrial Science & Technology (RIST) Pohang Gyeongbuk Republic of Korea; ^4^ Department of Materials Science and Engineering Pohang University of Science and Technology Pohang Gyeongbuk Republic of Korea

**Keywords:** dehydration, moisture stability, prussian blue, sodium‐ion batteries, surface oxidation

## Abstract

Na‐rich Prussian Blue (PB) is an attractive sodium‐ion battery cathode owing to its high capacity and low cost, yet approximately 10 wt.% of crystal water in its framework induces electrolyte side reactions and Fe dissolution. Conventional thermal dehydration has been used to remove crystal water, but it consistently results in capacity fading and poor air stability. Here, the primary cause of degradation is experimentally demonstrated to be surface oxidation driven by Fe–O bond formation during heat treatment. Guided by this insight, a liquid‐phase, low‐temperature dehydration strategy based on nitrogen bubbling is introduced, which simultaneously eliminates crystal water and stabilizes the surface. The approach can be seamlessly integrated into electrode fabrication to minimize air exposure and improve cycling stability. Overall, this work clarifies the mechanistic role of surface oxidation in moisture‐sensitive PB cathodes and highlights the critical importance of surface chemistry control in achieving stable electrochemical performance.

## Graphical abstract

Gas bubbling in a liquid medium lowers the local water vapor pressure and continuously removes desorbed water, enabling effective dehydration of Prussian Blue without inducing surface oxidation. This interfacial mass transfer‐driven process stabilizes the surface chemistry and improves electrochemical performance, providing a strategy for handling moisture‐sensitive Prussian Blue materials.

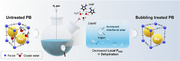

## 1 Introduction

Sodium‐ion batteries (SIBs) have attracted increasing attention as next‐generation energy storage systems because they employ sodium, an abundant and low‐cost resource, thereby overcoming the resource limitations of lithium [1, 2]. The performance and capacity of SIBs are primarily determined by cathode materials, with layered oxides [3], polyanionic compounds [4, 5], and prussian blue (PB) [6, 7] representing the most widely studied families. Among them, PB has gained significant interest due to its open three‐dimensional framework, which facilitates the insertion and extraction of large Na^+^ ions. Also, PB is highly cost‐effective, relying on inexpensive iron as the main component, and its synthesis is simple. Unlike layered oxide cathodes that require high‐temperature calcination [8, 9], PB is readily synthesized by mixing precursor solutions containing sodium and iron salts [10, 11] or through self‐assembly of a single precursor under acidic conditions [12, 13], which minimizes energy consumption during processing. Despite these advantages, PB suffers from intrinsic crystal water within its framework, which gives rise to a variety of challenges [14]. Typically comprising 10–20 wt.%, crystal water not only reduces the effective capacity in proportion to its mass but also accelerates electrolyte side reactions [15], promotes rapid capacity fading [16] and gas evolution [17], and impedes Na^+^ transport [18]. Therefore, controlling crystal water is essential for the practical application of PB as SIB cathodes. The origin of crystal water lies in the aqueous synthesis route, where rapid crystallization traps water molecules inside the framework [19]. Strategies such as introducing chelating agents [20] or employing co‐solvents [21, 22, 23, 24] have been reported, but the residual water content remains at ∼10 wt.%, indicating that the fundamental issues remain unresolved. Another approach has been to suppress the electrochemical activity of water rather than removing it, for example through electrolyte additives [25] or by leveraging water‐capture reactions [26, 27] that immobilize water molecules. While such strategies improve cycling stability, they inevitably sacrifice capacity because a fraction of Na^+^ ions remains electrochemically inactive.

Consequently, extensive efforts have been made to completely remove crystal water from the PB framework to achieve a higher specific capacity. Several studies have reported thermal dehydration under inert atmospheres at elevated temperatures (180–350°C) to eliminate crystal water from the structure. Upon dehydration, enhanced Na^+^ mobility and improved gravimetric capacity have been observed. For instance, it has been shown that crystal water removal can induce the formation of a new trigonal phase, thereby improving cycling stability [16]. Similarly, other reports demonstrated that dehydration activates the low‐spin Fe sites, which remain inactive in hydrated PB, thus enabling full utilization of both Fe redox couples [18, 28, 29]. Although high‐temperature thermal dehydration has been widely studied, the severe capacity fading of dehydrated PB at high voltages has often been ascribed to structural factors such as lattice collapse or volume changes, and ultimately regarded as an intrinsic limitation of the material itself. By contrast, the role of surface oxidation, particularly Fe─O bond formation during heat treatment, has not been sufficiently emphasized. Recent studies have revealed that such Fe─O bonds formed during cycling accelerate Fe dissolution, resulting in irreversible capacity loss and electrode degradation [30]. Moreover, surface Fe(OH)_3_ is thermodynamically unstable, thereby facilitating moisture adsorption and severely compromising air stability. Consistently, when hydrated and dehydrated PB particles were exposed to controlled‐humidity atmospheres, the dehydrated samples exhibited accelerated oxygen uptake and lattice contraction, indicating that rehydration and surface degradation can proceed rapidly [31, 32]. These processes proceed within 30 min of air exposure, which not only hinders uniform electrode slurry preparation but also leads to significant degradation in cathode performance. These findings demonstrate that conventional thermal dehydration inherently promotes Fe─O bond formation and surface oxidation, which fundamentally limit the durability of PB electrodes. The removal of structural water inevitably induces the oxidation of surface Fe species, even under inert or vacuum atmospheres, thereby revealing the inherent limitations of conventional thermal dehydration. Consequently, the development of alternative strategies that can eliminate crystal water while suppressing surface degradation is highly desirable for advancing the practical application of PB‐based electrodes.

In this work, we propose a novel liquid‐phase dehydration process that overcomes the limitations of conventional high‐temperature heat treatments, which inevitably induce surface degradation. Unlike non‐aqueous synthesis routes (Table S1), this work does not modify the PB synthesis itself. It targets crystal water removal after aqueous synthesis by replacing conventional solid‐state heat treatment with a liquid‐phase process. This method effectively removes crystal water while suppressing Fe─O bond formation. Notably, even after conventional high‐temperature heat treatment for crystal water removal, a measurable amount of gas evolution (Figure 4h) is still observed during the first charge, indicating that thermal dehydration alone does not fully eliminate surface‐driven parasitic reactions. Specifically, as‐synthesized PB containing crystal water is dispersed in the non‐aqueous solvent N‐methyl‐2‐pyrrolidone (NMP), followed by nitrogen bubbling at a relatively low temperature to remove crystal water. Continuous nitrogen bubbling reduces the partial pressure of oxygen and facilitates the outward diffusion of water molecules, thereby suppressing surface oxidation during dehydration. As a result, the formation of Fe─O bonds is markedly inhibited, and the reduced Fe^2^^+^ state is better preserved compared to conventional heat treatment. Furthermore, since NMP is the same solvent typically employed in electrode fabrication, this process offers the practical advantage of integrating dehydration with electrode preparation. Overall, the proposed liquid‐phase dehydration strategy minimizes surface degradation compared with conventional heat treatments while ensuring compatibility with electrode manufacturing. This approach is expected to improve both the initial performance and long‐term cycling stability of PB cathodes, providing a new pre‐treatment pathway for the broader utilization of crystal water‐containing PB.

## 2 Results & Discussion

### 2.1 Na Content Effects and Limitations of Thermal Dehydration in PB Cathodes

**FIGURE 1:**
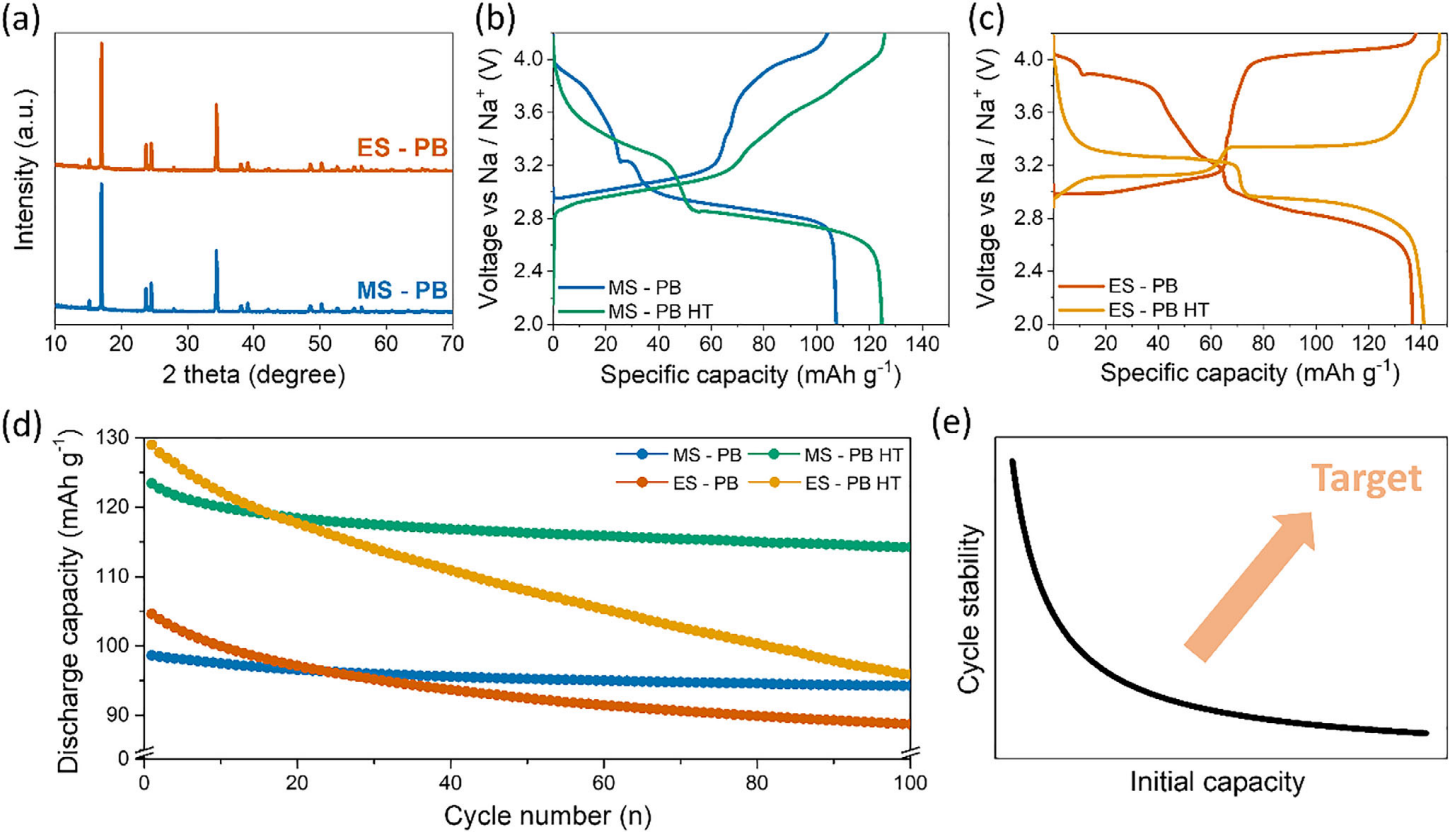
Electrochemical behavior of sodium‐rich PB with varying sodium content (a) XRD patterns of ES‐PB and MS‐PB, (b,c) Voltage profiles of (b) MS‐PB and MS‐PB HT, (c) ES‐PB and ES‐PB HT at the first cycle, (d) Cycling performance of MS‐PB, ES‐PB, and their HT counterparts, (e) Conceptual diagram of the targeted balance between capacity and stability.

Prussian Blue (PB) possesses an open three‐dimensional framework that provides ample space for Na^+^ storage. In general, Na‐intercalated PB can be expressed as Na_x_Fe[Fe(CN)_6_] (0 < x ≤ 2), where the electrochemical behavior strongly depends on the intrinsic Na content. Although Na‐rich PB phases have been widely reported, their charge–discharge profiles and accessible capacities vary substantially across studies, even when similar average crystallographic features are observed (Figure 1a). To clarify the role of intrinsic Na content, we synthesized two PB compositions with distinct Na levels, moderate‐sodium PB (MS‐PB) and elevated‐sodium PB (ES‐PB) (SI1), and further compared them with their heat‐treated (HT) counterparts in which crystal water was removed. These samples showed pronounced differences in charge–discharge behavior, voltage profiles, and reversible capacity. These results indicate that even within Na‐rich PB, variations in intrinsic Na content can lead to distinct electrochemical responses.

As shown in the first charge–discharge profiles (Figure 1b,c), ES‐PB delivered a higher initial capacity than MS‐PB and exhibited reduced polarization after heat treatment. However, ES‐PB suffered from severe structural instability and rapid capacity fading due to the large amount of Na^+^ participating during cycling (Figure 1d). This behavior is consistent with prior findings that excessive Na^+^ insertion induces lattice volume fluctuations, ultimately compromising cycle life [27]. While low‐Na PB variants have been explored to improve structural robustness, practical applications still demand sufficiently high initial capacities, which inherently require elevated Na content. Thus, rather than restricting Na content, strategies are needed to preserve high Na content while ensuring structural stability, as schematically illustrated in Figure 1e.

Building on these insights, this study focuses on the design of PB cathodes capable of combining high initial capacity with enhanced cycling stability. Although dehydration at high Na content can extend Na^+^ diffusion pathways and enlarge storage volume, it simultaneously exacerbates lattice volume changes and structural instability, leading to side reactions such as framework collapse and gas evolution. Nevertheless, prior studies have largely emphasized capacity maximization through dehydration, with limited attention to stability. Indeed, many reported voltage profiles show parasitic reactions above 4 V or negligible capacity improvements despite claims of complete dehydration, with little discussion of the underlying mechanisms. These observations highlight the intrinsic limitations of thermal dehydration and establish the starting point of this work. In particular, heat‐treated PB samples exhibit strong surface signatures of Fe─O bonding, which accelerate oxidation and moisture re‐adsorption, ultimately deteriorating material integrity (Figure 4e). Yet, the direct correlation between such surface changes and long‐term electrochemical stability has remained insufficiently addressed. Here, we focus on ES‐PB to systematically probe the role of surface oxidation in electrode degradation and to establish a liquid‐phase dehydration strategy that mitigates these effects.

### 2.2 Verifying Crystal Water Removal Through Bubbling‐Assisted Dehydration

**FIGURE 2:**
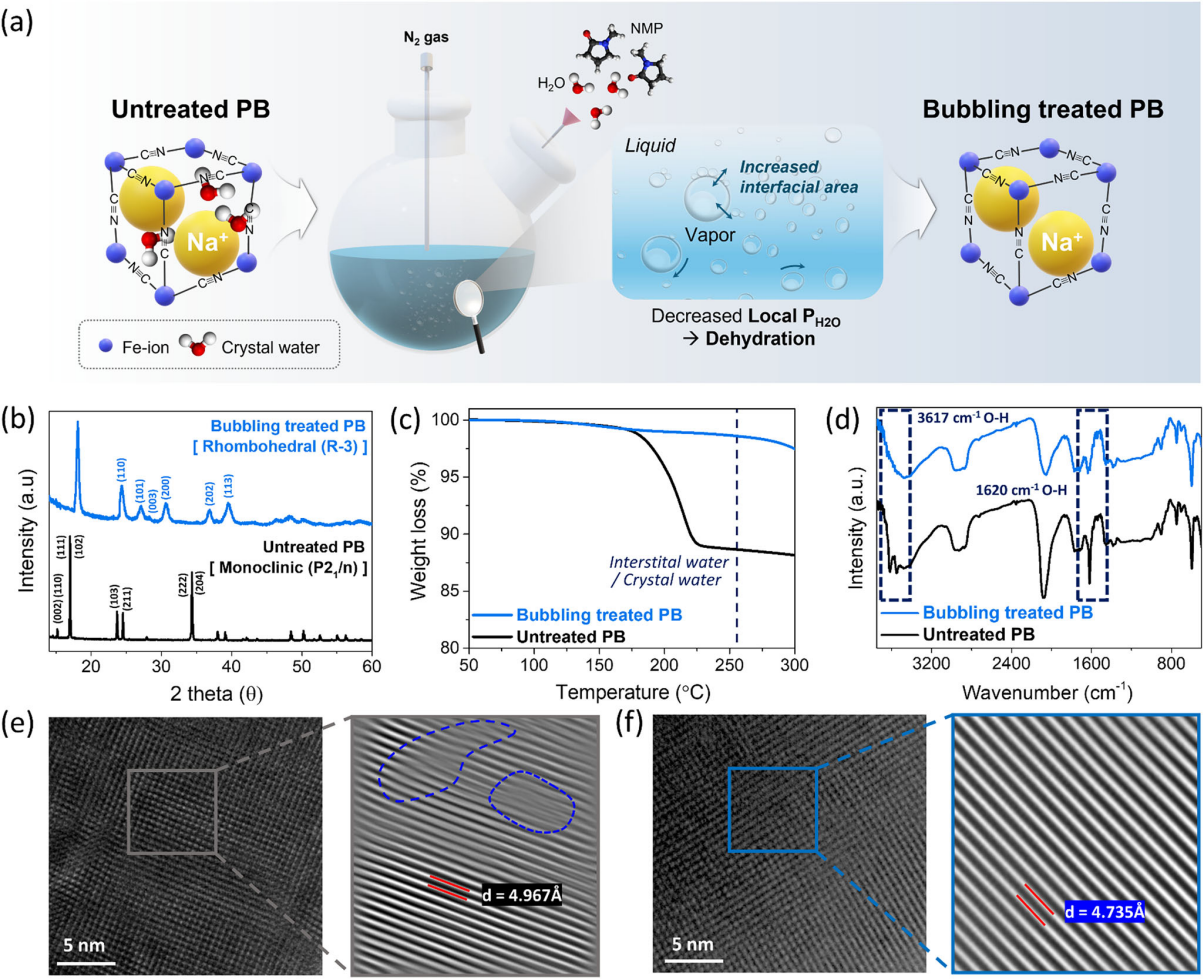
Validation of bubbling treatment strategy for eliminating crystal water: (a) Schematic illustration of bubbling‐induced dehydration via reduced local vapor pressure under an inert atmosphere, (b) XRD patterns, (c) TGA data, (d) FTIR data of untreated PB and bubbling treated PB, (e, f) HRTEM images and corresponding inverse fast Fourier transform (IFFT) lattice‐fringe images of (e) untreated PB and (f) bubbling‐treated PB showing the lattice spacing change after treatment.

To suppress surface oxidation during dehydration, we dispersed synthesized ES‐PB in the non‐aqueous solvent N‐methyl‐2‐pyrrolidone (NMP) and continuously purged nitrogen gas to maintain Fe in the divalent state. The bubbling process was carried out at a relatively mild temperature of 80 °C, where nitrogen purging accelerated the evaporation of NMP, thereby simultaneously promoting the removal of crystal water from the PB framework (SI2). Importantly, this dehydration behavior cannot be explained by temperature or solvent choice alone. Continuous N_2_ bubbling generates a dynamic gas–liquid interface that maintains a low water partial pressure, preventing equilibrium saturation. Under static conditions, water that reaches the particle surface is not efficiently removed and therefore accumulates. This accumulation rapidly diminishes the internal–surface chemical potential difference, causing the net dehydration rate to become negligible. In contrast, N_2_ bubbling continuously removes desorbed water and prevents surface accumulation. As a result, the chemical potential gradient across the particle is preserved, sustaining diffusion without altering the intrinsic diffusion properties of the solid (Figure 2a).

Fourier Transform Infrared Spectroscopy (FTIR) analysis of the solvent collected at the outlet reveals a pronounced O─H stretching band (∼3500 cm^−^^1^) that is absent in pristine NMP, directly confirming that crystal water released from PB is transferred into the NMP phase and removed together with the evaporating solvent (SI3). In contrast, control experiments conducted at the same temperature without N_2_ bubbling show negligible NMP evaporation and only limited crystal‐water removal, while iron oxidation continues to progress (SI4). These results demonstrate that the solvent presence alone is insufficient and that dynamic nitrogen bubbling is the key driving force enabling effective low‐temperature dehydration. This low‐temperature liquid‐phase route was then systematically evaluated to verify its effectiveness in eliminating crystal water through a series of structural and spectroscopic analyses. X‐ray diffraction (XRD) analysis shows systematic differences between untreated PB and nitrogen bubbling–treated PB (Figure 2b). The untreated PB containing crystal water can be described by an average monoclinic (P2_1_/n) structure, whereas the bubbling‐treated PB exhibits diffraction features consistent with those reported for dehydrated Na‐rich PB and can be described by an average rhombohedral (R‐3) symmetry. This average structure is consistent with previously reported dehydrated PB phases, which are also known to be accessible through conventional thermal dehydration, indicating that the observed structural evolution is associated with crystal‐water removal rather than a distinct synthesis‐dependent effect [33]. Thermogravimetric Analysis (TGA) results further revealed that the crystal water content decreased from ∼12 wt.% in untreated PB to ∼1 wt.% after bubbling, while FTIR spectra showed that the characteristic O─H vibration peaks associated with adsorbed and structural water at ∼1620 and ∼3617 cm^−^^1^, respectively, were significantly weakened following the treatment (Figure 2c,d). These results collectively verify the effective removal of crystal water from the PB framework through the bubbling process.

To further investigate structural changes at the lattice scale, High Resolution Transmission Electron Microscopy (HRTEM) analysis was performed (Figure 2e,f). A slight reduction in lattice spacing was observed after dehydration, indicating structural contraction upon water removal. Untreated PB exhibited blurred lattice fringes and local defects or dislocations, likely associated with the presence of crystal water, whereas the bubbled PB sample displayed well‐defined lattice fringes, confirming an ordered structure after dehydration [15]. These observations demonstrate that the bubbling process is an effective and reliable approach to eliminate crystal water from PB.

### 2.3 Electrochemical Effects of Crystal Water Removal and Low‐Spin Fe Activation

**FIGURE 3:**
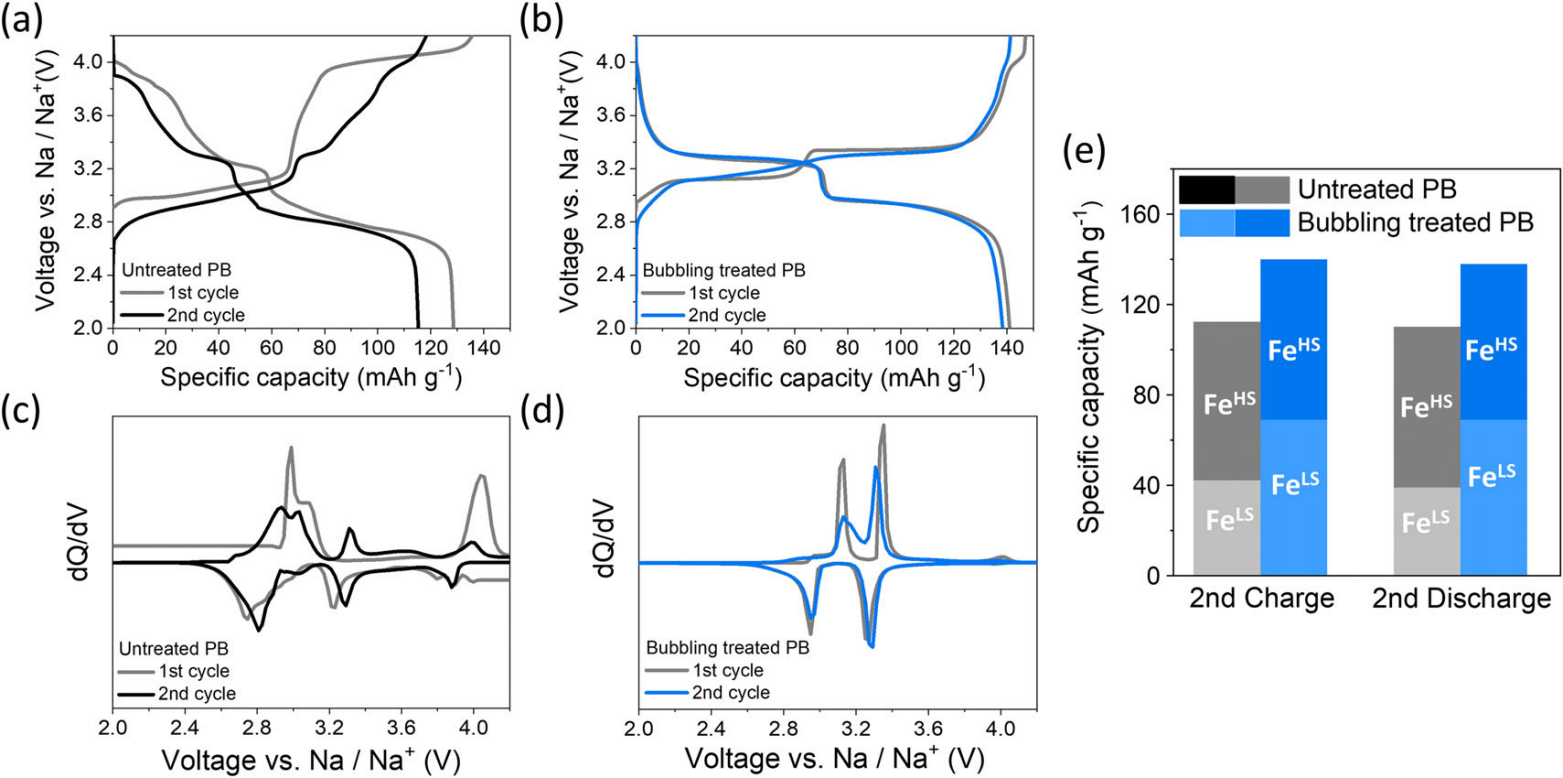
Voltage–capacity profiles for the first two cycles of (a) untreated and (b) bubbling‐treated PB. dQ/dV plots of (c) untreated and (d) bubbling‐treated PB. (e) Specific capacity contribution of high‐spin and low‐spin Fe states during the second cycle, derived from charge/discharge profiles.

To evaluate the effect of crystal water removal on the electrochemical performance of sodium‐rich PB, galvanostatic cycling tests were conducted. The untreated PB, which contains crystal water, exhibited a rapid voltage rise between 3.2 and 3.9 V with little capacity contribution, followed by a distinct high‐capacity plateau near 4.0 V during the first cycle. In the second cycle, however, this 4.0 V plateau nearly disappeared, and the corresponding capacity contribution diminished during both charge and discharge. The initial capacity above 4.0 V is well known to originate from Na^+^ insertion/extraction at the 24d site and parasitic reactions triggered by structural water [23]. The fact that most of this capacity contribution vanished in the second cycle indicates that these processes are largely irreversible, consistent with water‐driven side reactions (Figure 3a).

In contrast, the bubbled PB showed clear suppression of irreversible capacity above 4.0 V, along with the emergence of an additional capacity contribution around 3.2–3.3 V (Figure 3a). Previous studies have reported that dehydration activates low‐spin Fe sites, which are electrochemically inactive in the hydrated state, thereby providing extra capacity [18, 29]. Accordingly, removal of crystal water not only suppresses irreversible parasitic reactions in the first cycle but also enhances the overall specific capacity through the activation of low‐spin Fe sites. These features are more explicitly confirmed in the differential capacity (dQ/dV) plots (Figure 3c,d).

To quantitatively evaluate the contribution of low‐spin Fe activation, capacity analysis was performed for each plateau region. To exclude irreversible contributions from water decomposition during the initial cycle, the second cycle at 0.1C (1C = 140 mAh g^−^^1^) was used as the basis for analysis. As shown in Figure 3e, the electrochemical contribution from low‐spin Fe ions was significantly enhanced after dehydration, confirming that crystal water removal enables the activation of previously inactive Fe sites.

This interpretation is further supported by the evolution of the dQ/dV profiles. In untreated PB, the voltage region associated with low‐spin Fe (∼3.2–3.3 V) is barely visible during the first cycle, but a weak feature emerges from the second cycle onward (SI7). This behavior indicates that the presence of crystal water suppresses low‐spin Fe redox activity in the initial cycle. Consistently, Galvanostatic intermittent titration technique (GITT) analysis further reveals larger overpotentials in this voltage region for untreated PB, supporting the kinetic limitation of low‐spin Fe in the hydrated state (S18).

### 2.4 Suppressing Surface Oxidation and Gas Evolution Through Liquid‐Phase Bubbling Dehydration

**FIGURE 4:**
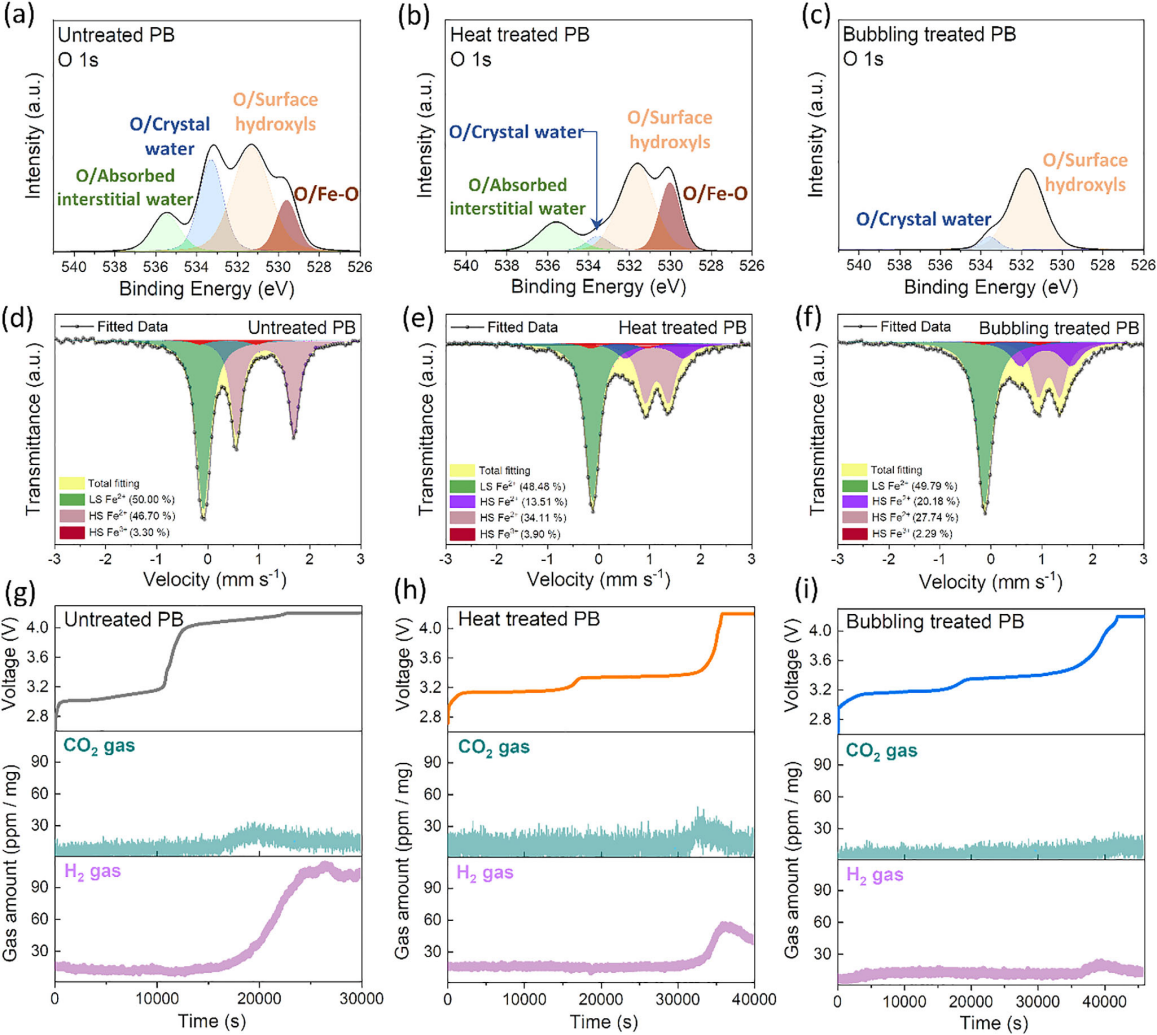
Surface and oxidation‐state characterization of PB samples: (a–c) O 1s XPS spectra of (a) untreated, (b) heat‐treated, and (c) bubbling‐treated PB, (d–f) Room‐temperature ^5^ ^7^Fe Mössbauer spectra of (d) untreated, (e) heat‐treated, and (f) bubbling‐treated PB, (g–i) In situ DEMS analysis showing gas evolution profiles during the first charge for (g) untreated, (h) heat‐treated, and (i) bubbling‐treated PB.

This study further highlights the distinction and advantages of the liquid‐phase dehydration process compared with conventional high‐temperature heat treatment. When PB is dehydrated through thermal annealing, crystal water is expelled from the framework and can promote the formation of Fe─O bonds at the particle surface (Figure 4b). These Fe─O bonds are thermodynamically unstable and tend to evolve into oxidized iron species, leading to an increased Fe^3^^+^ fraction. In contrast, PB treated by the bubbling process suppressed the formation of additional Fe─O bonds under an inert atmosphere (Figure 4c). This behavior originates from continuous inert gas purging, which maintains low partial pressures of oxygen and water while facilitating the removal of desorbed species from the interface. As a result, whereas conventional heat treatment leads to partial oxidation of Fe and a decrease in the Fe^2^^+^ fraction, the liquid‐phase bubbling process instead favors the preservation of Fe^2^^+^ species.

The change in Fe oxidation state was also indirectly reflected in the color evolution during the bubbling treatment. PB is known to exhibit a lighter blue appearance (approaching Prussian White) when enriched in Fe^2^^+^, whereas a deep blue color is associated with Fe^3^^+^‐dominant states. During the bubbling process, the suspension color gradually shifted from deep blue to sky blue over 6 h (SI2), consistent with partial reduction of Fe species. This visual observation supports the notion that the liquid‐phase process effectively suppresses oxygen adsorption and surface oxidation during crystal water removal.

To determine whether this stabilization extends beyond the surface, Mössbauer spectroscopy was employed as a bulk‐sensitive probe. The Mössbauer spectra revealed that heat‐treated PB exhibits an increased Fe^3^^+^/Fe^2^^+^ ratio, indicative of oxidation during thermal dehydration (Figure 4e). In contrast, the bubbled PB maintained a higher Fe^2^^+^ fraction within the bulk lattice, confirming that the liquid‐phase process preserves the reduced iron state throughout the framework (Figure 4f).

The suppression of parasitic reactions was further corroborated by in situ differential electrochemical mass spectrometry (in situ DEMS) during first charging (Figure 4g–i). The untreated PB containing crystal water exhibited substantial gas evolution at high potentials, attributable to water‐induced side reactions. Although heat treatment reduced the overall gas intensity, a considerable amount of gas evolution persisted, which, in light of the preceding analyses, is reasonably attributed to oxidized oxygen species remaining at the particle surface. In sharp contrast, the bubbled PB showed strongly suppressed gas evolution under identical conditions. This result indicates that simultaneously controlling bulk crystal water and surface oxygen species is critical for mitigating gas‐evolving side reactions, demonstrating the effectiveness of the liquid‐phase bubbling strategy in stabilizing the electrode–electrolyte interface.

Collectively, these results demonstrate that the liquid‐phase bubbling treatment provides a dehydration pathway that not only removes crystal water under oxygen‐free conditions but also stabilizes the Fe^2^^+^ state in the bulk lattice and suppresses gas‐evolving side reactions during charging. This fundamentally differentiates the bubbling process from conventional heat treatment.

### 2.5 Electrochemical Performance Evaluation of Bubbling‐Treated PB Cathodes

**FIGURE 5:**
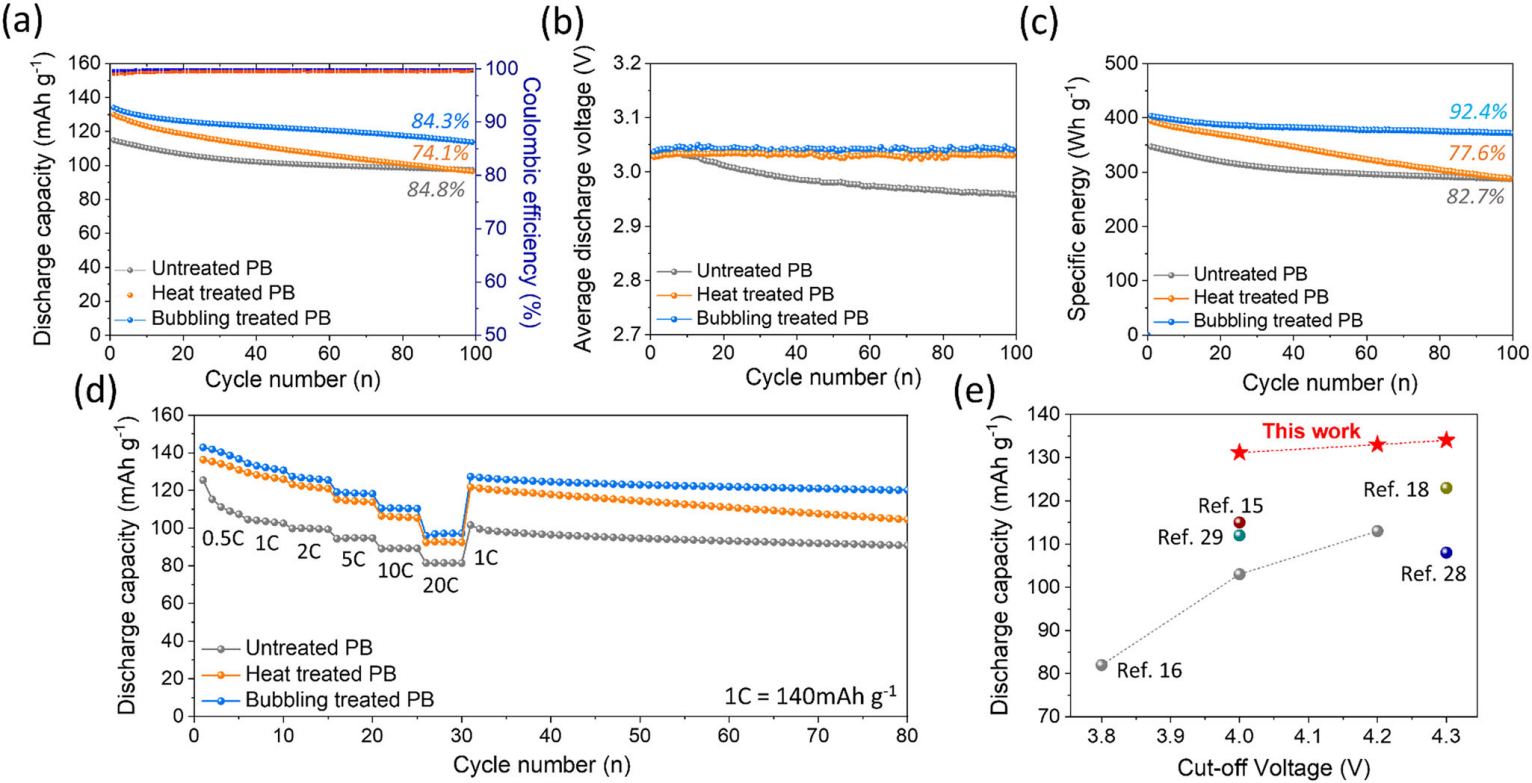
Electrochemical performance evaluation of PB‐based materials: (a) Discharge capacity, (b) average discharge voltage, and (c) average energy (calculated as capacity × voltage). (d) Rate capability at various current densities. (e) Comparative performance with previously reported PB‐based materials.

The electrochemical performance of PB samples containing crystal water and those dehydrated by either heat treatment or bubbling was systematically evaluated. The heat‐treated sample was optimized to achieve a comparable level of crystal water removal to the bubbled sample, while avoiding excessively harsh conditions that could induce bulk structural degradation and compromise capacity (SI6, SI7). At a current density of 1C over 100 cycles, both dehydrated samples exhibited higher specific capacities than the hydrated PB, confirming the beneficial effect of crystal water removal. However, the heat‐treated sample showed a lower capacity retention of 74.1%, whereas the bubbled sample maintained 84.3% of its initial capacity, underscoring the superior cycling stability achieved through the liquid‐phase process (Figure 5a). Consistent with this trend, over 100 cycles the bubbling‐treated PB maintained the highest and most stable Coulombic efficiency (CE), while the untreated and heat‐treated samples exhibited CE deterioration associated with water‐ or surface‐induced irreversible reactions (SI13). These results further highlight the importance of simultaneously controlling crystal water and surface chemistry to sustain long‐term reversibility.

In addition to capacity retention, the preservation of average discharge voltage is a critical factor in determining the overall energy density of a cell. Unlike the hydrated PB, which suffered rapid voltage decay due to side reactions and resistance buildup, both dehydrated samples exhibited stable voltage profiles. As a result, the energy retention (capacity × average voltage) was highest for the bubbled sample (Figure 5c). Notably, although the hydrated PB displayed the highest capacity retention of 84.8% over 100 cycles, the superior voltage stability of the bubbled sample ultimately translated into the highest energy retention among all samples. Furthermore, crystal water removal also imparted improved rate performance, as demonstrated in Figure 5d.

To compare with previously reported heat‐treated PB cathodes, we evaluated the initial discharge capacities at 1C under comparable conditions, specifically with similar crystal water content and electrode loading (Table S2). While cycle stability varies significantly depending on the voltage window and capacity range, making direct comparison challenging, our samples consistently exhibited higher initial capacities across all tested voltage ranges (Figure 5e). This superior performance can be attributed to the combined effects of structural stabilization and minimized surface oxidation enabled by the bubbling process. Taken together, these findings demonstrate that the proposed strategy enables the design of PB cathodes that achieve both high initial capacity and long‐term stability, consistent with the ideal balance illustrated in Figure 1e.

To further elucidate the origin of the improved cycling behavior, the evolution of redox reactions was examined using dQ/dV analysis. The untreated PB shows negligible features in the low‐spin Fe–related voltage region (3.2–3.3 V) during early cycles, whereas both dehydrated samples exhibit pronounced and persistent peaks from the first cycle, indicating that crystal water suppresses low‐spin Fe redox activity while its removal enables a stable contribution to capacity (SI14). The structural and interfacial stability during prolonged cycling was evaluated by Scanning Electron Microscopy (SEM) analysis. After 100 cycles, the sodium metal electrode paired with untreated PB exhibits a rough and highly non‐uniform reaction layer, whereas the bubbling‐treated PB maintains a more uniform and compact surface morphology, reflecting effective suppression of water‐derived parasitic reactions (SI15). Consistently, post‐cycling SEM images of PB particles reveal severe surface degradation for untreated PB, pronounced cracking for heat‐treated PB, and a higher fraction of structurally intact particles for bubbling‐treated PB (SI16). Electrochemical impedance spectroscopy further supports these observations. The bubbling‐treated PB electrode exhibits a smaller semicircle after formation and maintains the lowest overall impedance after 100 cycles, indicating mitigated interfacial resistance and suppressed impedance growth during long‐term cycling (SI17). To further probe the interfacial chemical evolution, Time‐of‐Flight Secondary Ion Mass Spectrometry (TOF‐SIMS) analysis was conducted after 100 cycles. The CO_3_^−^ signal, attributed to carbonate species formed via reactions between oxygen‐containing species and the electrolyte, is significantly reduced in the bubbling‐treated sample compared to the untreated and heat‐treated samples (SI18).

### 2.6 Moisture Adsorption and Surface Reactivity in Dehydrated PB Electrodes

**FIGURE 6:**
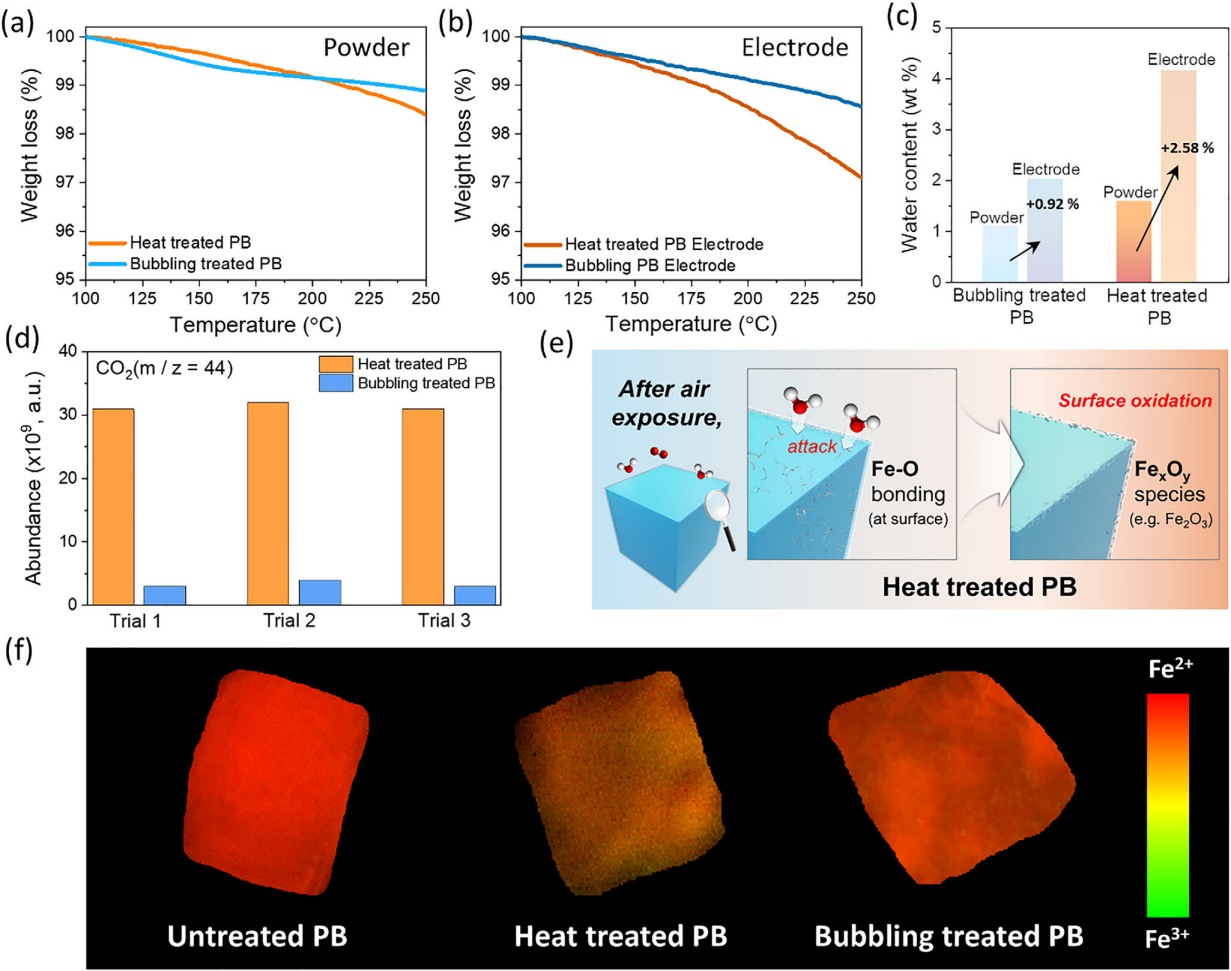
Evaluation of air stability and post‐cycling reactivity of PB samples: TGA profiles of (a) powder and (b) electrode, (c) Summary of moisture content from two samples. (d) GC‐MS analysis of CO_2_ from cycled electrodes soaked in fresh electrolyte, (e) Proposed mechanism of surface oxidation during heat‐driven dehydration, (f) Fe K‐edge XANES imaging of each sample after 30 min exposure (RH 20%).

Both heat‐treated PB and bubbling‐treated PB contained ∼1–2 wt.% residual water after dehydration (Figure 6a). However, distinct differences emerged during electrode processing. During 30 min to 1 h of electrode slurry preparation, the heat‐treated PB absorbed significantly more moisture than the bubbled PB (Figure 6b). Quantitative analysis revealed an increase of 2.58 wt.% for the heat‐treated PB, compared with only 0.92 wt.% for the bubbled PB (Figure 6c), highlighting that Fe─O bonds on the surface of the heat‐treated sample act as favorable sites for moisture adsorption. Additional powder‐level exposure experiments were conducted at a relative humidity of 20% to further differentiate the intrinsic moisture affinity of the samples (SI19). After 1 h of air exposure, the mass loss below ∼170°C, corresponding to surface‐adsorbed water, was consistently higher for the heat‐treated PB than for the bubbling‐treated PB, indicating a higher susceptibility to moisture uptake associated with surface oxidation.

Post‐mortem analysis after 100 cycles further underscored this effect. The separator from the heat‐treated cell exhibited pronounced brown discoloration, whereas no visible staining was observed for the bubbling‐treated sample (SI20), indicating more extensive Fe migration in the heat‐treated case. Inductively Coupled Plasma (ICP) analysis of electrolytes also revealed an Fe concentration of 0.743 ppm for the heat‐treated PB, compared to 0.305 ppm for the bubbling‐treated PB under identical conditions. Together, the visual separator evidence and quantitative ICP results consistently confirm that Fe dissolution and migration are significantly suppressed in the bubbling‐treated sample.

To probe the chemical stability of cycled PB electrodes, each sample was soaked in fresh electrolyte for 24 h, and the evolved gases were analyzed by Gas Chromatography–Mass Spectrometry (GC‐MS). This test was designed to assess whether surface by‐products or reactive species formed during cycling remained active, with CO_2_ generation used as an indirect measure of residual reactivity [34]. Triplicate experiments revealed consistently higher CO_2_ evolution from samples rich in Fe─O bonds (Figure 6d). These results indicate that Fe─O bonds serve as active sites that promote water adsorption, which subsequently reacts with electrolyte components to produce parasitic gases. Indeed, Fe─O bonds, owing to their strong oxygen affinity, readily capture trace moisture from air or electrolyte and retain it in bound form. Such water can further react with carbonate solvents (EC, DEC), generating CO_2_ and other decomposition gases, thereby compromising electrode stability. In contrast, the bubbling‐treated PB, with suppressed Fe─O bonding, exhibited significantly reduced CO_2_ release, clearly demonstrating its superior surface stabilization effect.

Based on these findings, a schematic illustration summarizing the surface oxidation pathway of the heat‐treated sample, in contrast to the bubbling‐treated sample, is presented in Figure 6e. To further validate this mechanism, single‐particle Fe K‐edge X‐ray absorption near‐edge structure (XANES) imaging [35] was performed after air exposure (RH 20%) for 30 min (Figure 6f). The heat‐treated PB exhibits a predominantly Fe^3^^+^‐like distribution across the particles, indicating rapid oxidation upon air exposure, whereas the bubbling‐treated PB shows significantly suppressed oxidation, confirming the effectiveness of the bubbling process in mitigating surface oxidation.

### 2.7 Integrated Dehydration–Fabrication Strategy for Practical PB Electrodes

**FIGURE 7:**
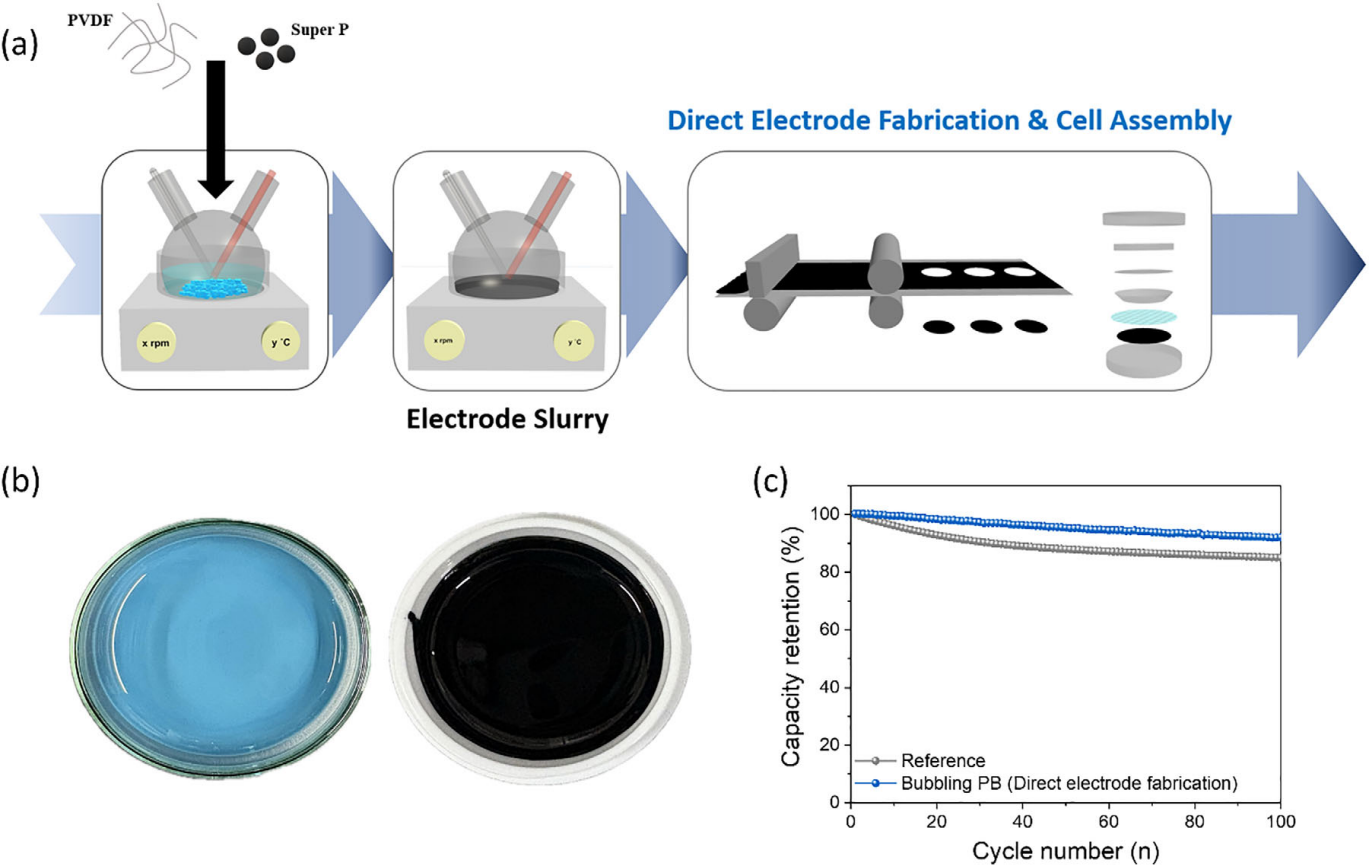
Direct electrode fabrication strategy using bubbling‐treated PB. (a) Schematic illustration of the integrated bubbling–slurry process. (b) Photographs of PB dispersed in NMP after bubbling (left) and the resulting electrode slurry after adding conductive agent and binder (right). (c) Electrochemical performance of the resulting electrode over 100 cycles.

While the bubbling‐based dehydration process effectively suppresses surface oxidation and mitigates electrode degradation, PB electrodes treated in this manner can still suffer from performance loss due to gradual moisture re‐adsorption during prolonged air exposure. To address this issue, we introduced a direct electrode fabrication strategy that integrates the liquid‐phase dehydration process with electrode preparation, thereby minimizing air exposure (Figure 7a). In this approach, dehydration and slurry formation are carried out sequentially under an inert atmosphere, simultaneously simplifying the process and improving moisture management. Notably, partial removal of the solvent during bubbling naturally adjusted the slurry viscosity, enabling uniform electrode fabrication without additional concentration‐control steps (Figure 7b).

Electrodes produced via this integrated strategy exhibited enhanced capacity retention and stable long‐term cycling performance compared with untreated samples, effects attributable to the removal of structural water and suppression of surface rehydration immediately following dehydration. These results highlight that controlling the post‐dehydration chemical environment is critical for maintaining the benefits of surface‐stabilized PB during electrode fabrication. Notably, the bubbling‐assisted dehydration process exhibits good reproducibility over a practical range of processing conditions, indicating its potential for scalable implementation. Furthermore, consistent effects were also observed for PB synthesized via an alternative method, suggesting that the proposed strategy is broadly applicable to crystal‐water‐containing PB systems.

## 3 Conclusion

In this work, we demonstrate that the conventional high‐temperature dehydration route for Na‐rich PB, while effective in removing crystal water, inevitably triggers surface oxidation through Fe─O bond formation. Such surface degradation not only accelerates capacity fading but also increases the susceptibility of the electrode to moisture uptake. To overcome this limitation, we developed a liquid‐phase, low‐temperature dehydration process based on nitrogen bubbling, which enables controlled removal of crystal water while simultaneously stabilizing the particle surface. Importantly, this process can be seamlessly integrated into electrode fabrication, thereby minimizing air exposure and eliminating the need for a dedicated drying environment, ultimately ensuring uniform electrode preparation. Building on these findings, this study clarifies that the electrochemical instability of Na‐rich PB is governed not only by the presence of crystal water but also by the surface chemical state established during dehydration. By comparing heat‐treated and bubbling‐treated PB, we demonstrate that suppressing Fe─O bond formation at the particle surface is critical for mitigating gas evolution, interfacial degradation, and long‐term performance decay. These results reveal that the dehydration pathway itself determines the surface reactivity and reproducibility of PB cathodes, highlighting post‐synthetic surface chemistry as a key design parameter. Overall, this work provides a mechanistic perspective on dehydration‐induced surface stabilization in moisture‐sensitive PB systems and emphasizes the importance of controlled surface chemistry in achieving stable and consistent electrochemical behavior.

## Conflicts of Interest

The authors declare no conflicts of interest.

## Supporting information




**Supporting File**: adma73507‐sup‐0001‐SuppMat.docx.

## Data Availability

All data supporting the findings of this study are available within the article and its supporting information.
