# Supplementary material for: Suppressing Surface Degradation in Na‐Rich Prussian Blue Cathodes via Liquid‐Phase Dehydration
Source: Adv Mater. 2026 May 27;38(36):e73507. doi: 10.1002/adma.73507 (PMC13310102; doi:10.1002/adma.73507)
Supplement: Supplementary file 1 — Supporting File: adma73507‐sup‐0001‐SuppMat.docx. [file ADMA-38-e73507-s001.docx]

Supporting Information

**Suppressing Surface Degradation in Na-Rich Prussian Blue Cathodes via Liquid-Phase Dehydration**

Seunghye Jang^a^, Hyebin Jeong^b^, Jun Choi^a^, Jeongsoo Hong^b^, Jooyoung Jang^a^, Jongyoon Han^c^, Juwon Kim^c^, Sang-Min Lee ^a, d^, and Changshin Jo ^a, b *^

a. Department of Battery Engineering,

Graduate Institute of Ferrous & Eco Materials Technology,

Pohang University of Science and Technology (POSTECH),

77 Cheongam-ro, Nam-gu, Pohang, Gyeongbuk, 37673, Republic of Korea

b. Department of Chemical Engineering,

Pohang University of Science and Technology (POSTECH),

77 Cheongam-ro, Nam-gu, Pohang, Gyeongbuk, 37673, Republic of Korea

c. Research Institute of Industrial Science & Technology (RIST),
67 Cheongam-ro, Nam-gu, Pohang, Gyeongbuk, 37673, Republic of Korea

d. Department of Materials Science and Engineering,

Pohang University of Science and Technology,

77 Cheongam-ro, Nam-gu, Pohang, Gyeongbuk 37673, Republic of Korea

* Corresponding authors.

E-mail: [jochangshin@postech.ac.kr](mailto:jochangshin@postech.ac.kr) (C. Jo)

**Experimental Section**

2.1. Materials preparation

For the synthesis of Prussian Blue (PB), sodium ferrocyanide decahydrate (Na₄Fe(CN)₆·10H₂O, Sigma-Aldrich) was employed as a single precursor. Hydrochloric acid (HCl, 37%, Duksan) was used to prepare the acidic solution, and trisodium citrate (Na₃C₆H₅O₇·2H₂O, Alfa Aesar) served as a chelating agent. All chemicals were used as received without further purification.

2.2. Synthesis of PB particles via acid-assisted self-assembly

PB was synthesized through precursor decomposition and reassembly in acidic solution. First, 0.2 M HCl (1.64 mL) was added to 200 mL of deionized water, followed by N₂ purging for 30 min to remove dissolved oxygen. Sodium ferrocyanide (0.02 M) and trisodium citrate (0.03 M) were then added, and the solution temperature was gradually increased to 80 °C. After reaching the target temperature, the suspension was stirred at 1000 rpm for 6 h and subsequently aged for an additional 12 h at room temperature. Continuous N₂ purging was maintained throughout the reaction to minimize Fe oxidation.
The resulting PB particles were collected by centrifugation and washed five times with 40 mL of a 1:1 (v/v) mixture of deionized water and ethanol at 8500 rpm. The washed samples were transferred to Petri dishes and vacuum-dried at 120 °C for 12 h to obtain the final PB powders.

2.3. Preparation of heat-treated PB via thermal treatment

The as-synthesized PB powder (0.2 g) was placed in a 5 mL vial and transferred to a vacuum oven, where a vacuum was applied. The temperature was then increased to 180 °C and maintained under vacuum for 6 h to remove crystal water from the PB framework. This temperature was optimized to ensure complete removal of crystal water without inducing excessive structural degradation. After the treatment, the dehydrated powder was rapidly transferred to an argon-filled glovebox for storage prior to use.

2.4. Bubbling treatment of self-assembled PB particles

As-synthesized PB (0.2 g) was dispersed in 20 mL of N-methyl-2-pyrrolidone (NMP) and stirred at 80 °C for 6 h. During the treatment, N₂ gas was continuously introduced through an inlet to suppress Fe oxidation, while solvent and crystal water were removed via an outlet stream. After bubbling, the suspension was cooled to room temperature, and the product was recovered and vacuum-dried at 120 °C for 12 h. The dried PB powders were immediately transferred into an Ar-filled glovebox to prevent structural changes and moisture adsorption caused by air exposure.

2.5. Electrochemical measurement

Electrodes were prepared by mixing PB powder (70 wt%), Super P (20 wt%), and PVDF binder (10 wt%) in NMP to form a homogeneous slurry. The slurry was cast onto Al foil, followed by drying at 120 °C for 12 h. The active material loading was approximately 2–3 mg cm⁻². CR2032 coin cells were assembled in an Ar-filled glovebox using Na metal as the counter/reference electrode, a glass fiber separator (GF/D), and 1 M NaPF₆ in EC/DEC (1:1 v/v) with 5 wt% FEC as the electrolyte. All electrochemical measurements were carried out at room temperature in an environmental chamber.

2.6. Materials characterization

**X-ray Diffraction (XRD)** patterns were recorded on a Bruker D8 Advance diffractometer using Cu Kα radiation (λ = 1.5406 Å) to determine the crystalline structure of the samples. **Scanning Electron Microscopy (SEM)** analysis was performed using a HITACHI S-4800 microscope to examine particle morphology and surface features. **Transmission Electron Microscopy (TEM)** images were obtained using a JEOL JEM-2200FS microscope operated at 200 kV for nanoscale structural analysis and to observe lattice fringes. **⁵⁷Fe Mössbauer spectroscopy** was conducted at room temperature to quantify the bulk Fe²⁺/Fe³⁺ ratios and to evaluate changes in the iron valence state after different dehydration treatments. **In-situ Differential Electrochemical Mass Spectrometry (DEMS)** was conducted to monitor gas evolution during electrochemical cycling. In-situ DEMS setup was directly coupled to the electrochemical cell to enable real-time detection of gaseous species generated during charge–discharge processes. Particle-level Fe oxidation-state distribution (i.e., Fe²⁺ and Fe³⁺) was investigated by TXM-based Fe K-edge XANES analysis (7C XNI beamline, Pohang Accelerator Laboratory). XANES image data were analyzed using the TXM-Pal software^34^ **Gas chromatography–mass spectrometry (GC–MS) was** performed to analyze gaseous by-products and confirm solvent removal after the bubbling treatment. **Inductively Coupled Plasma (ICP) Analysis** was performed to quantitatively evaluate Fe dissolution after electrochemical cycling. After cycling, the cells were carefully disassembled, and all electrolyte-contacted components were collected. The components were immersed in dimethyl carbonate (DMC) for 24 h to extract residual electrolyte species. The DMC was completely evaporated, and the remaining residue was dissolved and diluted in 1 wt% nitric acid (HNO) prior to ICP measurement.

**SI 1.** ICP data of MS-PB and ES-PB

(Unit: wt %)

|  | MS-PB | ES-PB |
| --- | --- | --- |
| Na | 10.0 | 12.5 |
| Fe | 28.3 | 29.8 |

**SI 2.** Optical image of bubbling treatment of PB: (a) immediately after bubbling, (b) after 6 h of bubbling


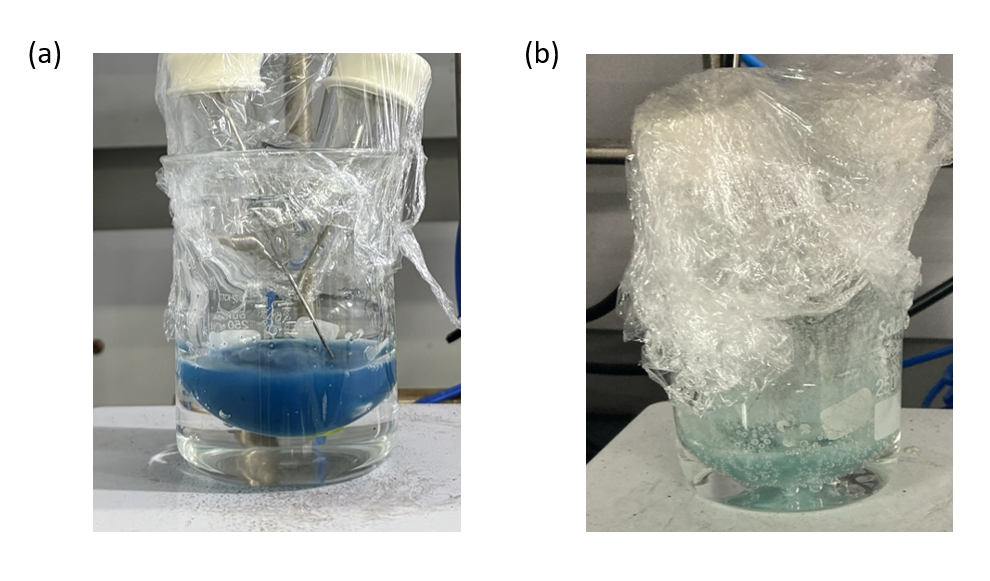


**SI 3.** FTIR spectra of pristine NMP and outlet-trapped solvent after N₂ bubbling


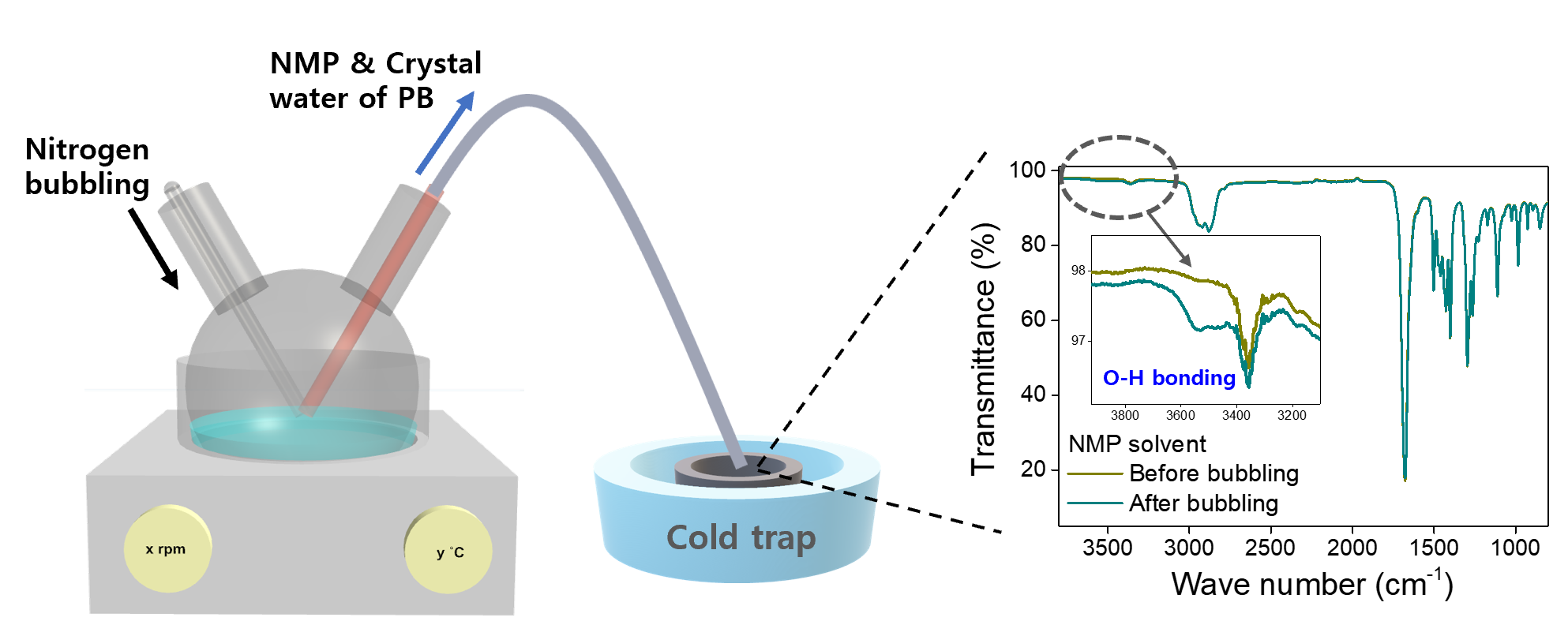


**SI 4.** (a) Optical image of the PB dispersion after static heating at 80 °C without N₂ bubbling; (b) XRD pattern of the powder obtained after the treatment.


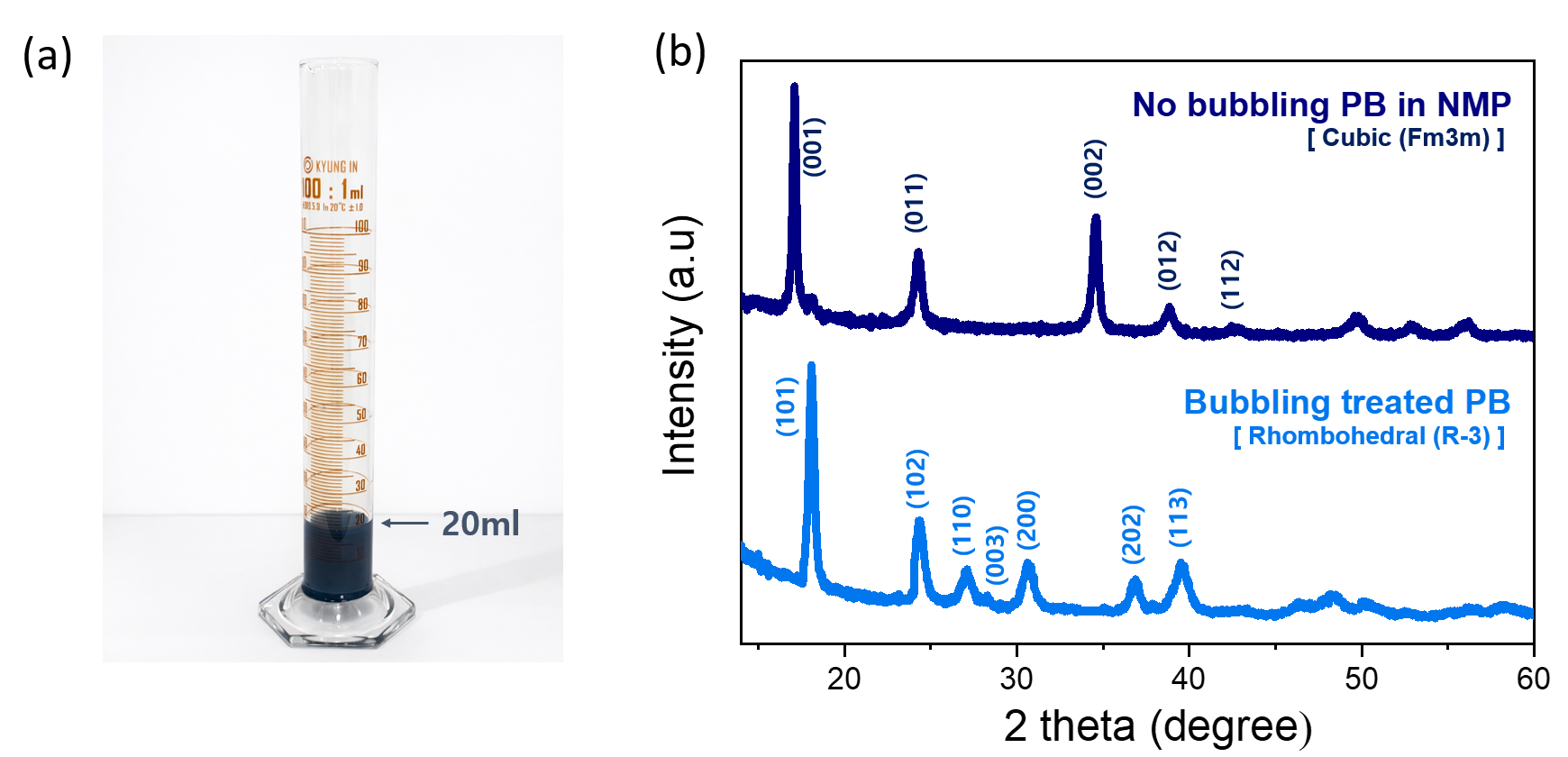


SI 5. In-situ heating XRD of crystal water-containing PB

**
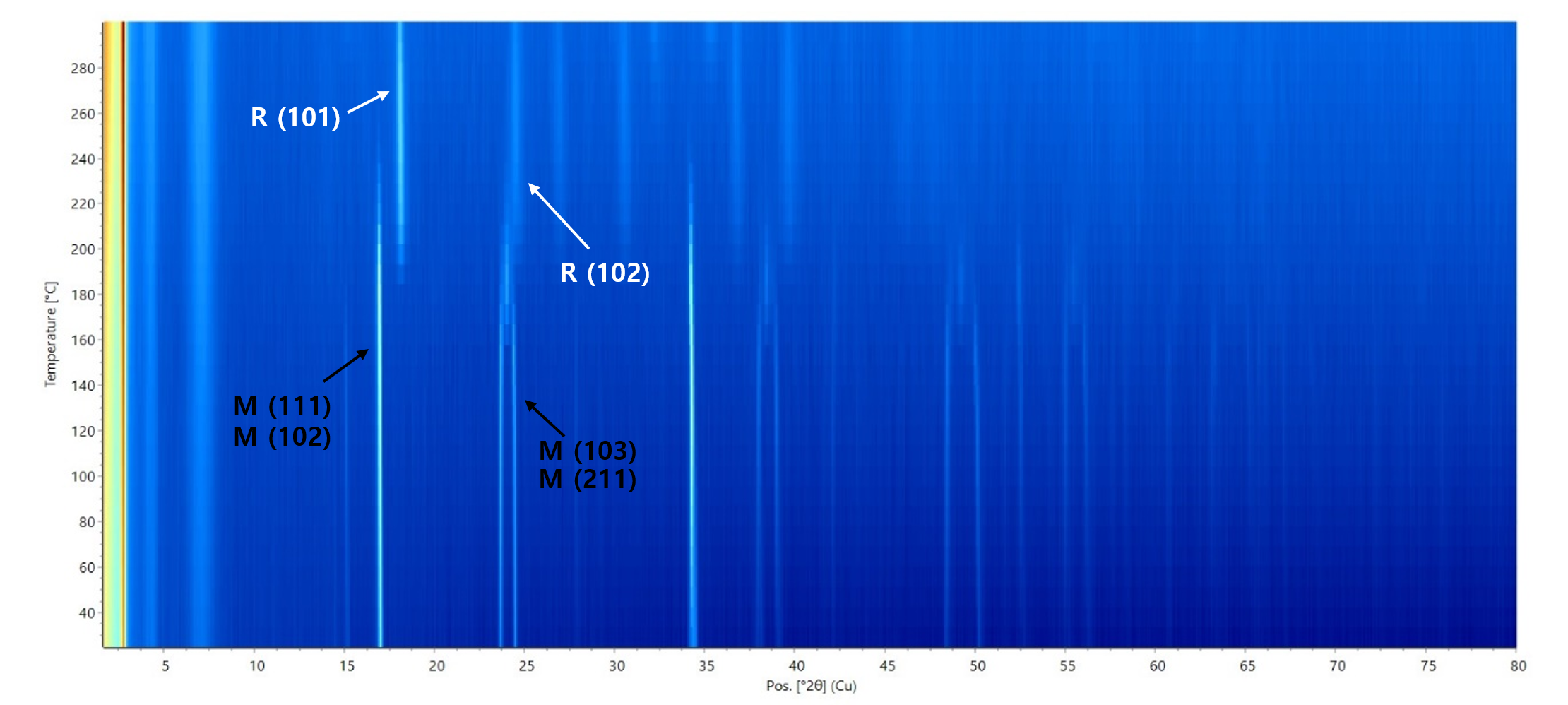
**

**SI 6.** XRD patterns of heat treated PB and Bubbling treated PB


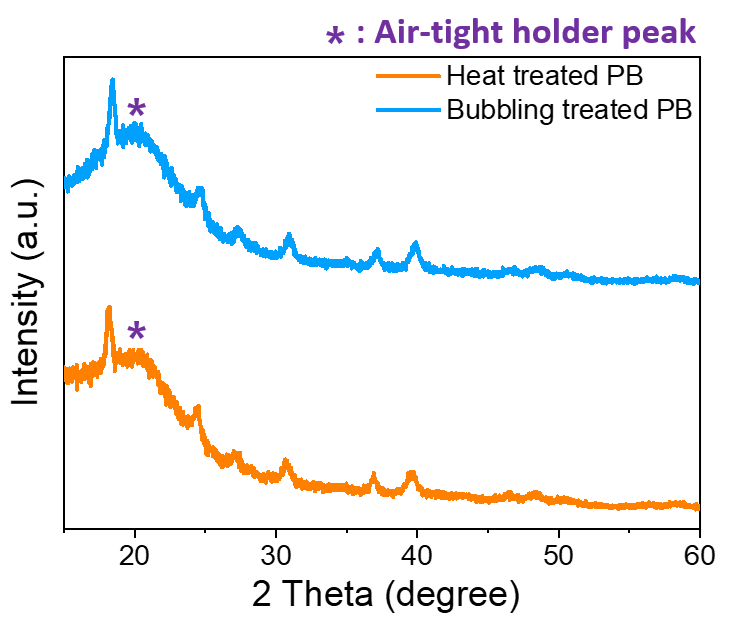


**SI 7.** Voltage profiles of heat treated PB at (a) 160˚C and (b) 200˚C


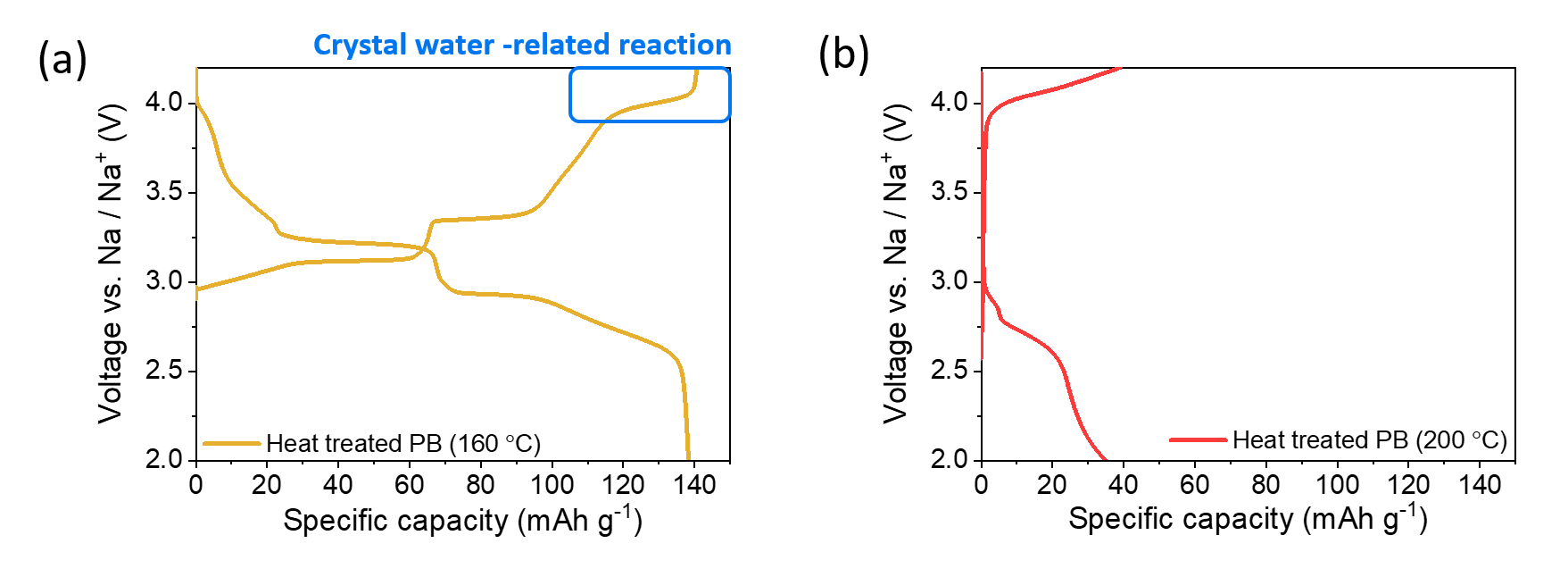


SI 8. dQ/dV profiles of untreated PB for the first and second charge–discharge cycles.


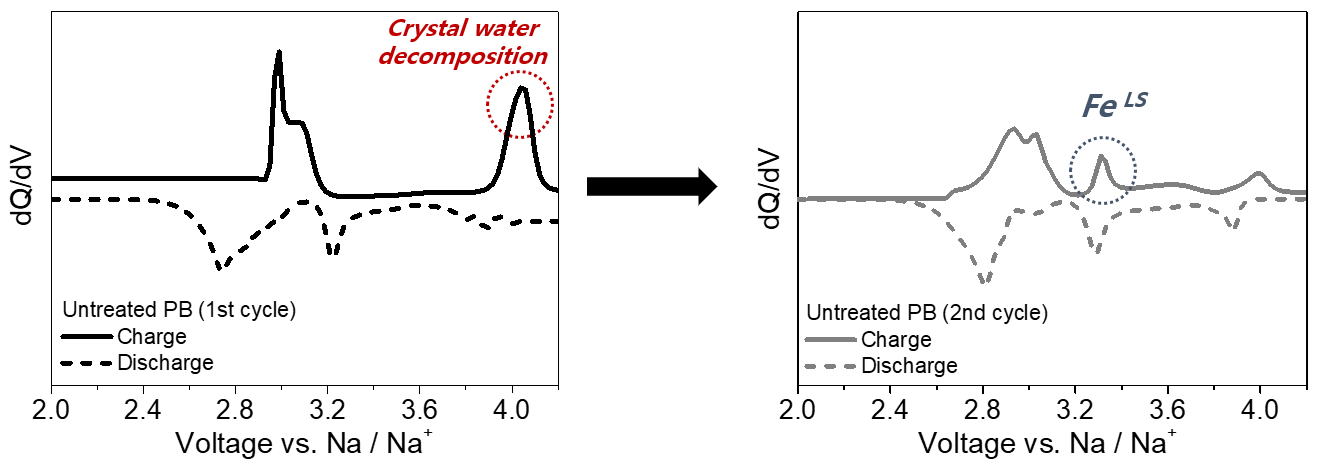


**SI 9.** GITT analysis of PB electrodes before and after crystal-water removal: (a) GITT profiles of untreated PB, (b) GITT profiles of bubbling-treated PB


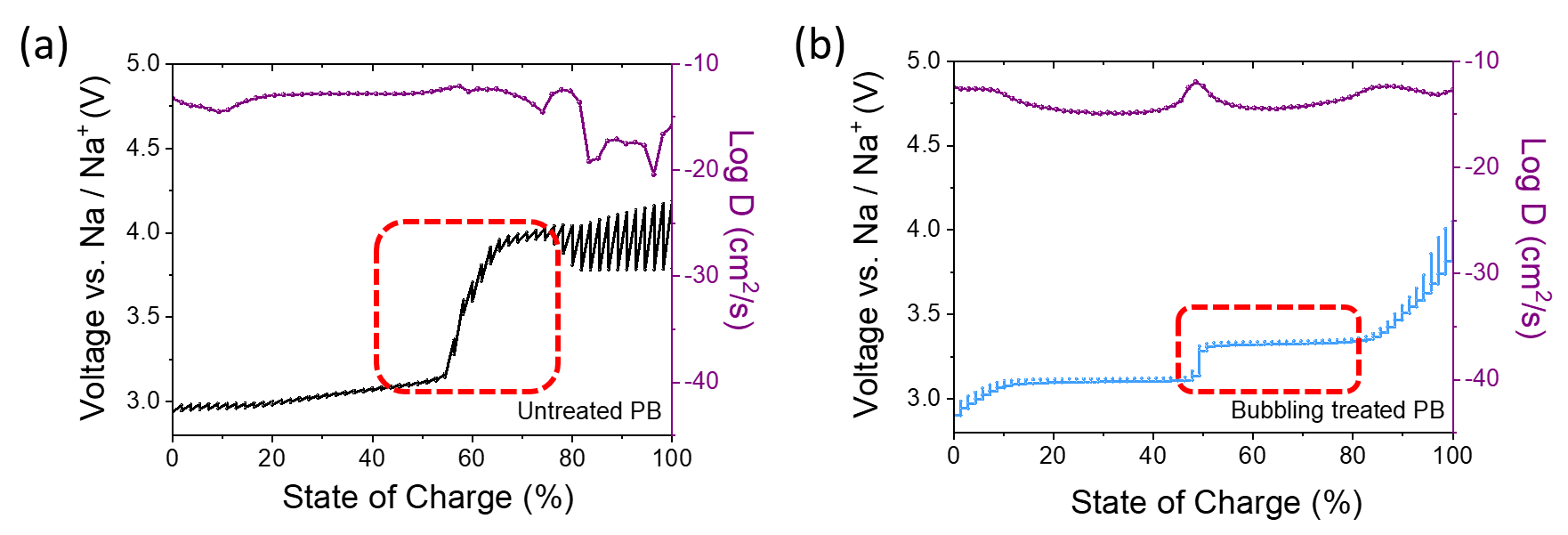


**SI 10.** Overpotential of untreated and bubbling-treated PB. (a) SOC-dependent profiles with Region 1 and 2. (b) Quantitative values.


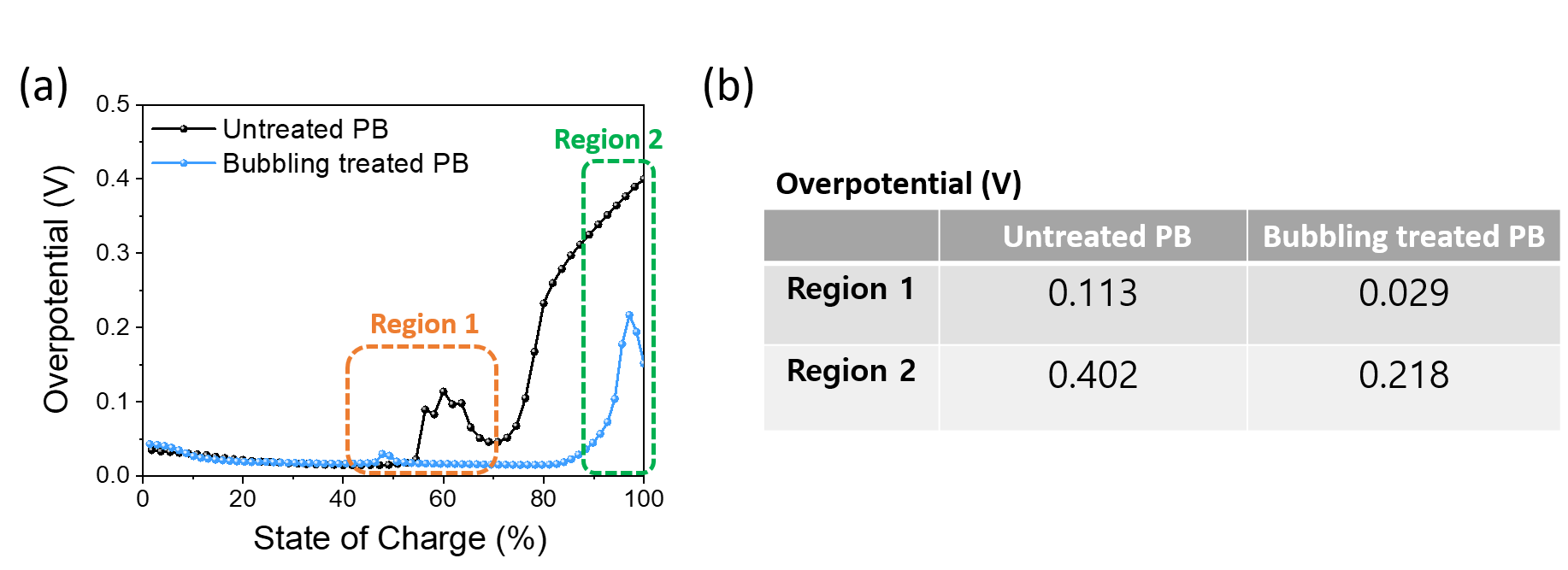


**SI 11.** Voltage profiles of (a) untreated and (b) bubbling-treated PB with local voltage hysteresis (ΔV) at the low-spin Fe region.


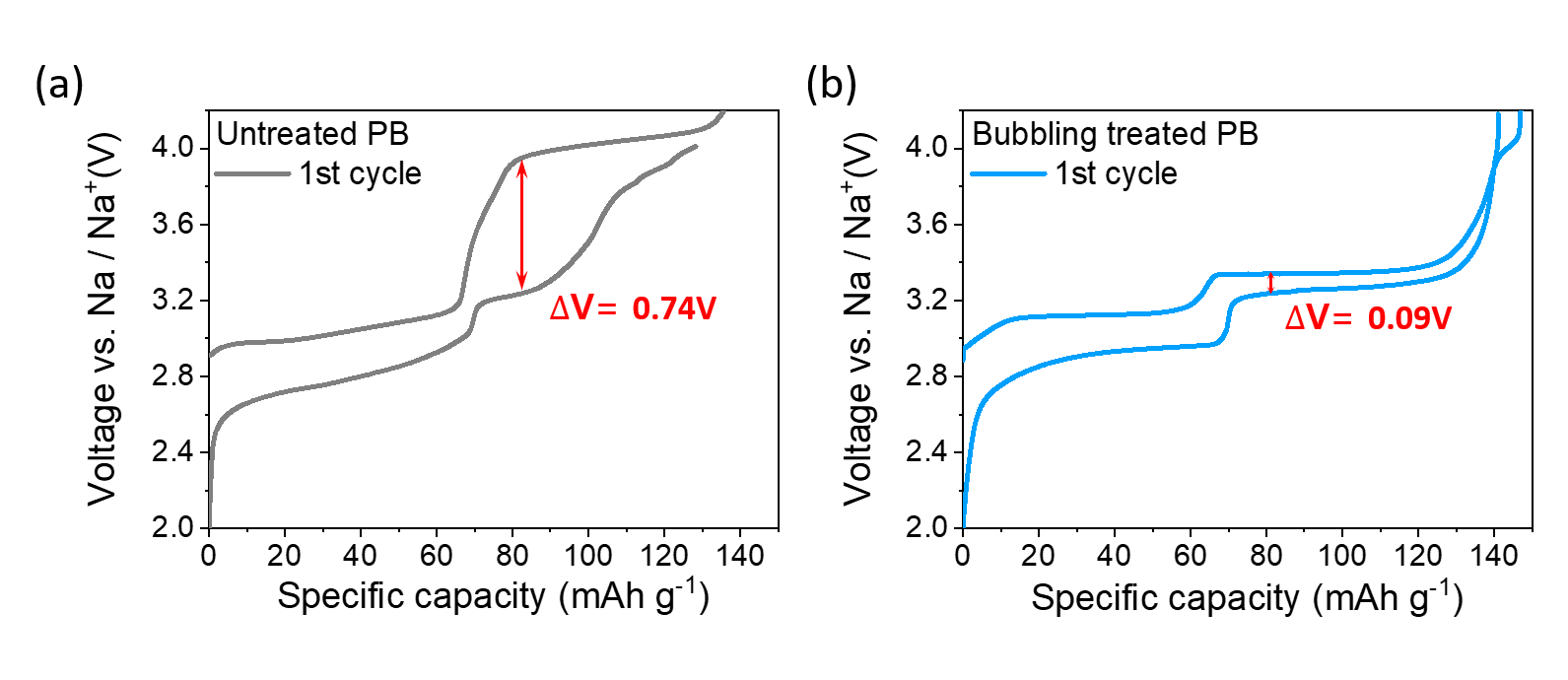


**SI 12.** Averaged cycling performance of PB electrodes from four independent coin cells.


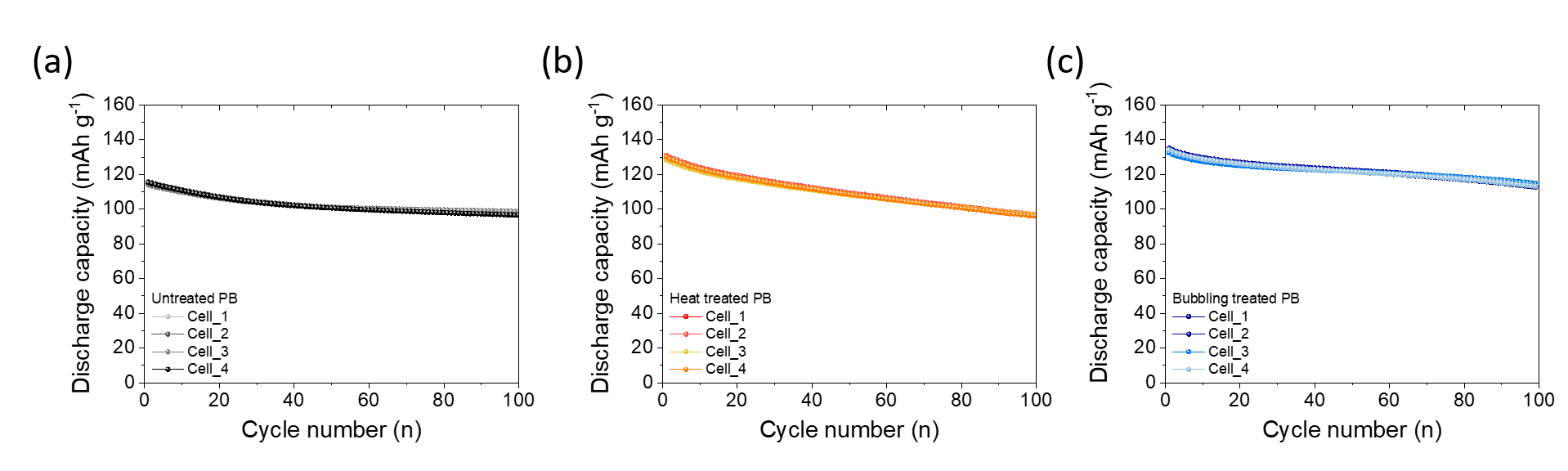


**SI 13.** Coulombic efficiency of each cell for 100 cycles


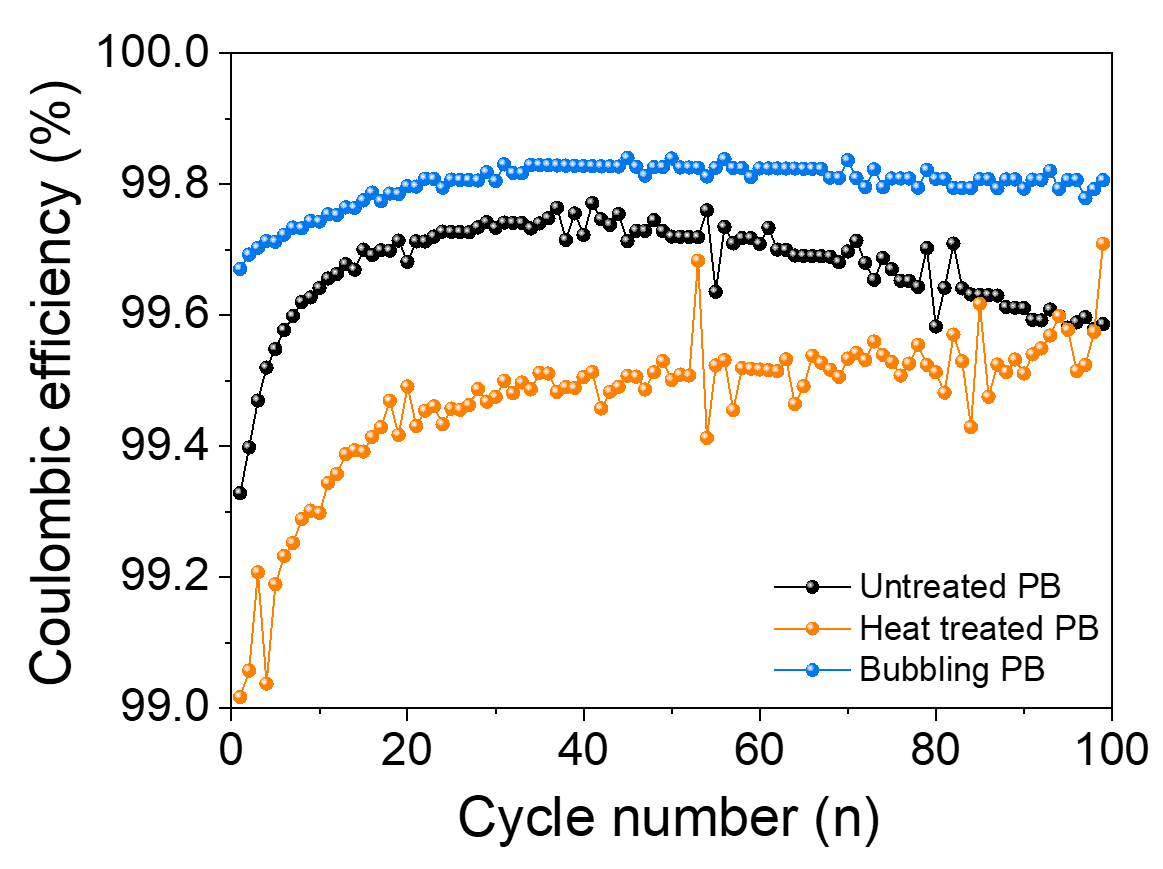


**SI 14.** Evolution of dQ/dV profiles of PB electrodes at the 5th, 50th, and 100th cycles of (a) Untreated PB, (b) Heat treated PB, (c) Bubbling treated PB


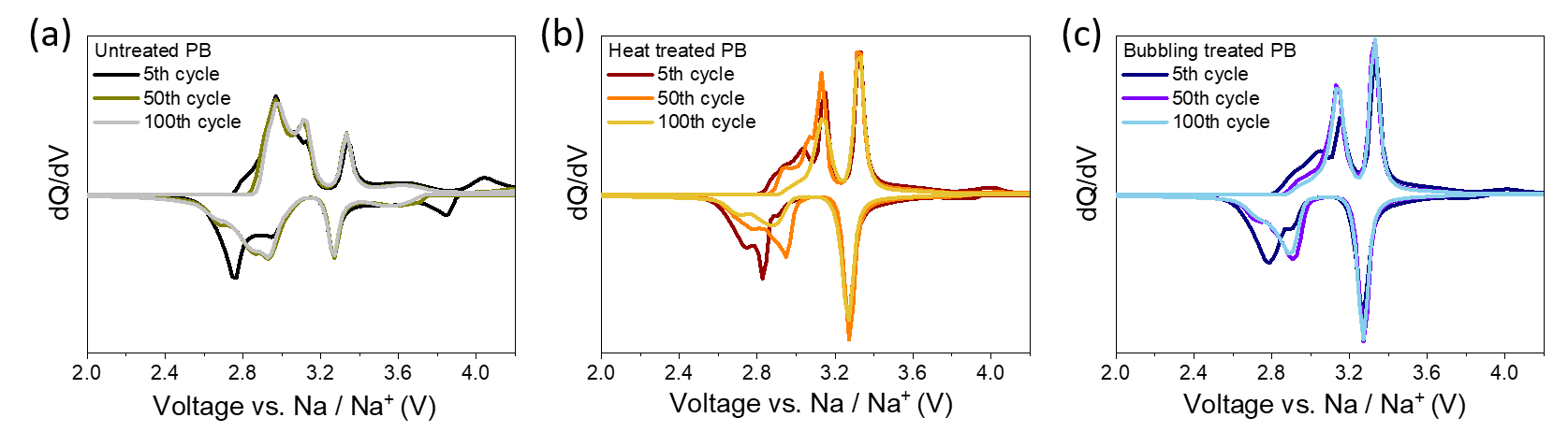
SI 15. SEM images of Na metal surfaces after the first charge in cells employing (a, b) untreated PB and (c, d) bubbling treated PB cathodes.


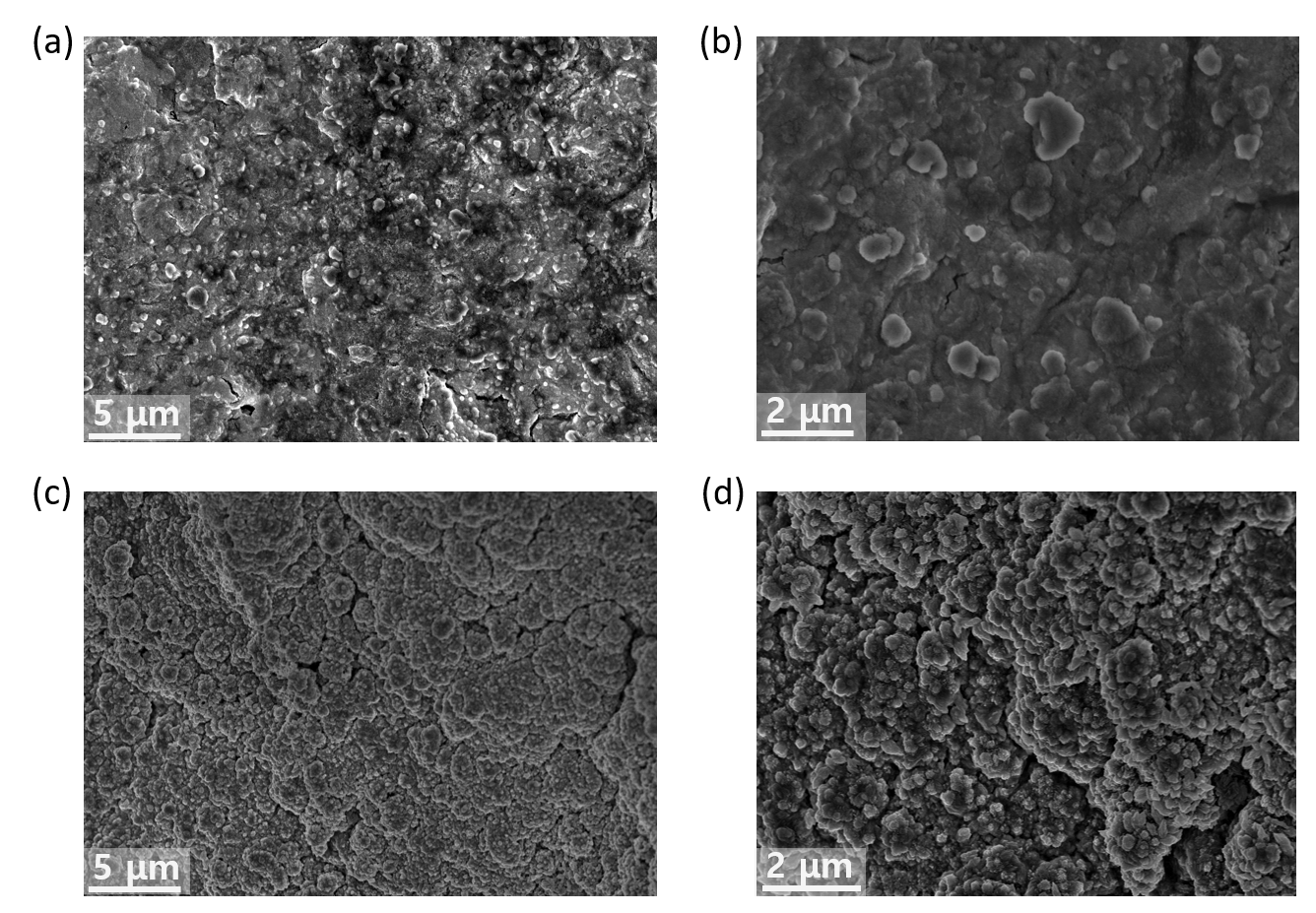


SI 16. SEM images of PB particles after 100 charge–discharge cycles


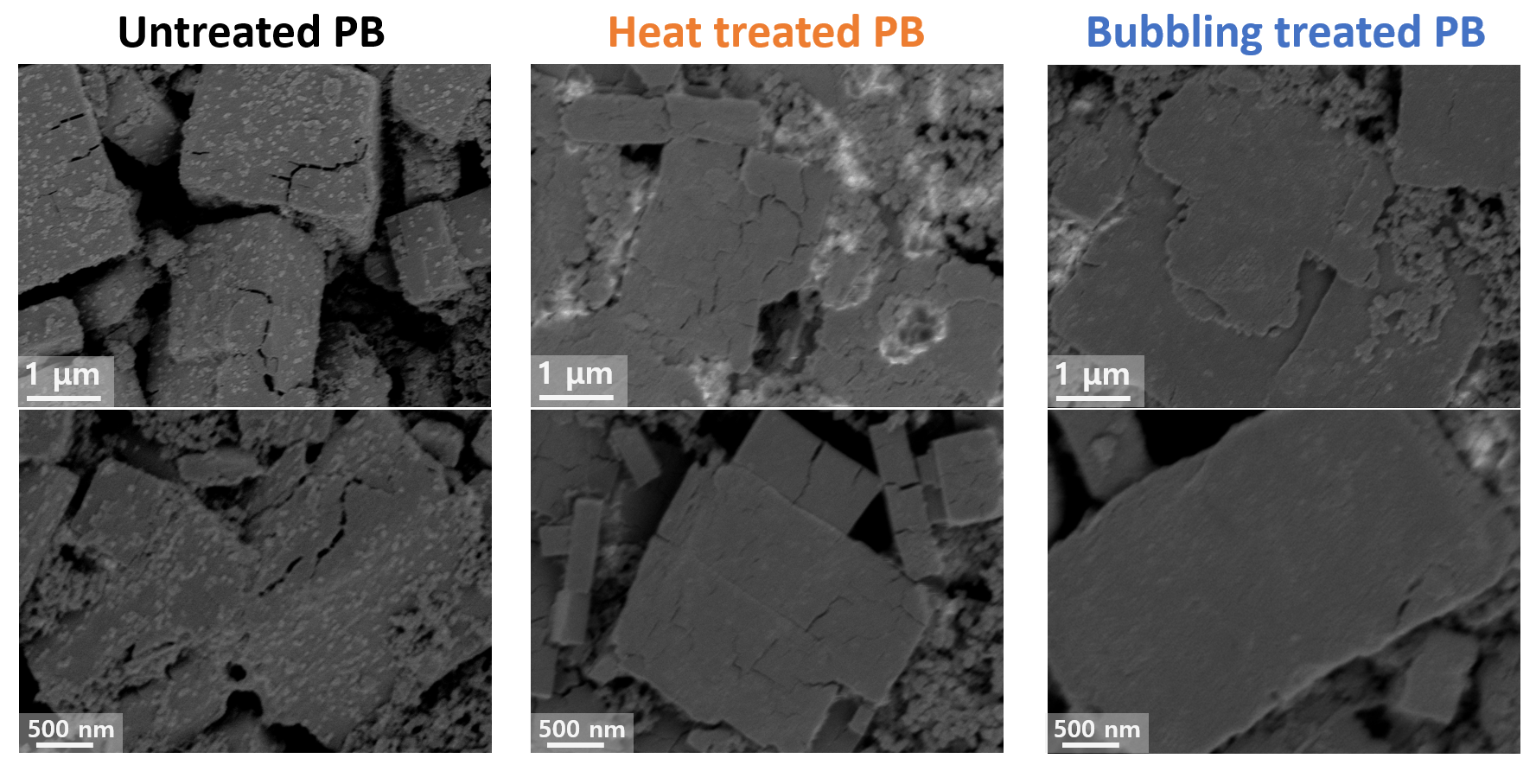


**SI 17.** EIS spectra after 1 cycle formation and (b) after 100 cycles (c) table of total resistance (R_total_).

**
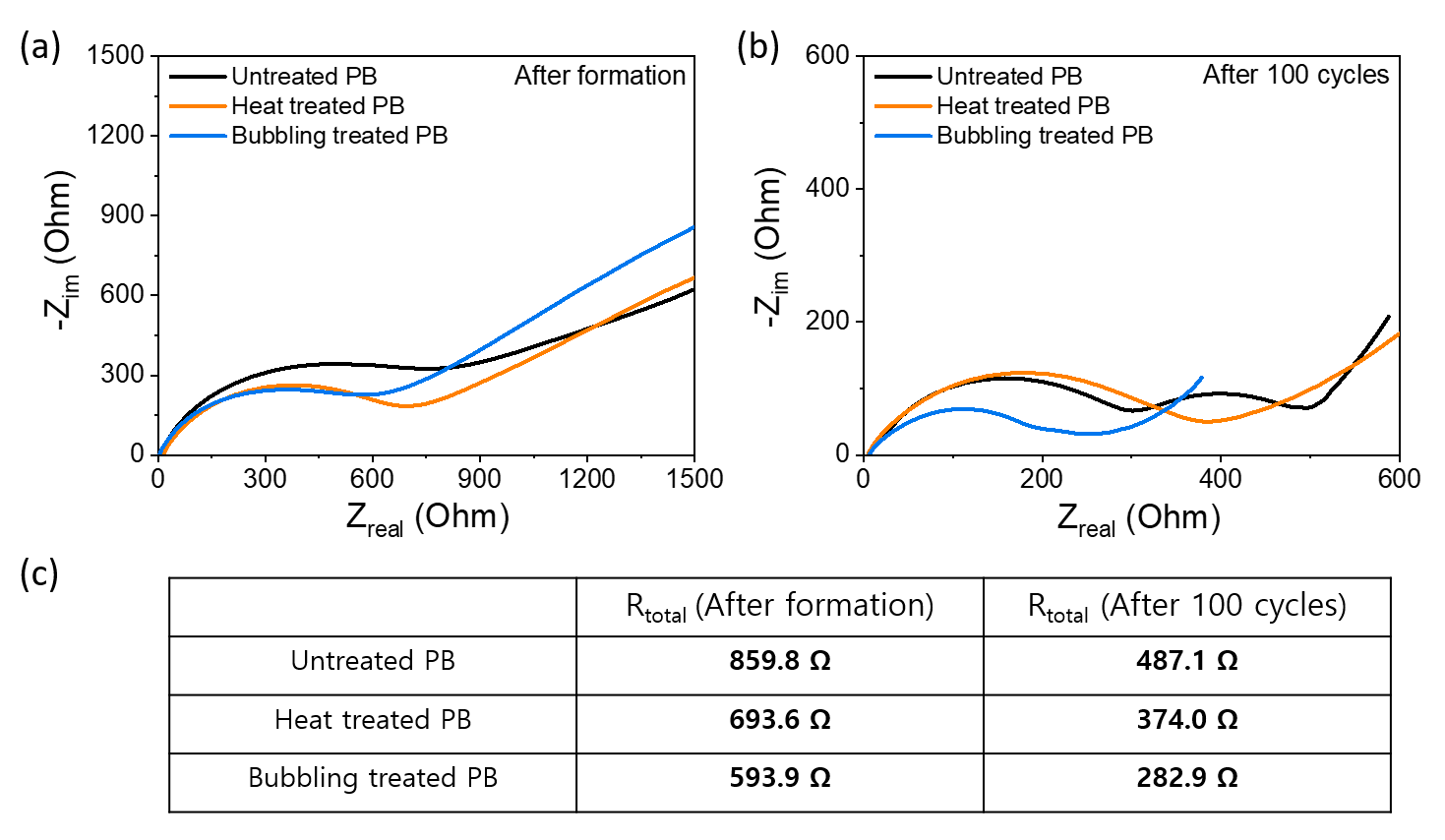
**

**SI 18**. TOF-SIMS results of PB electrodes after 100 cycles. (a–c) 2D CO₃⁻ ion distribution maps and (d–f) corresponding


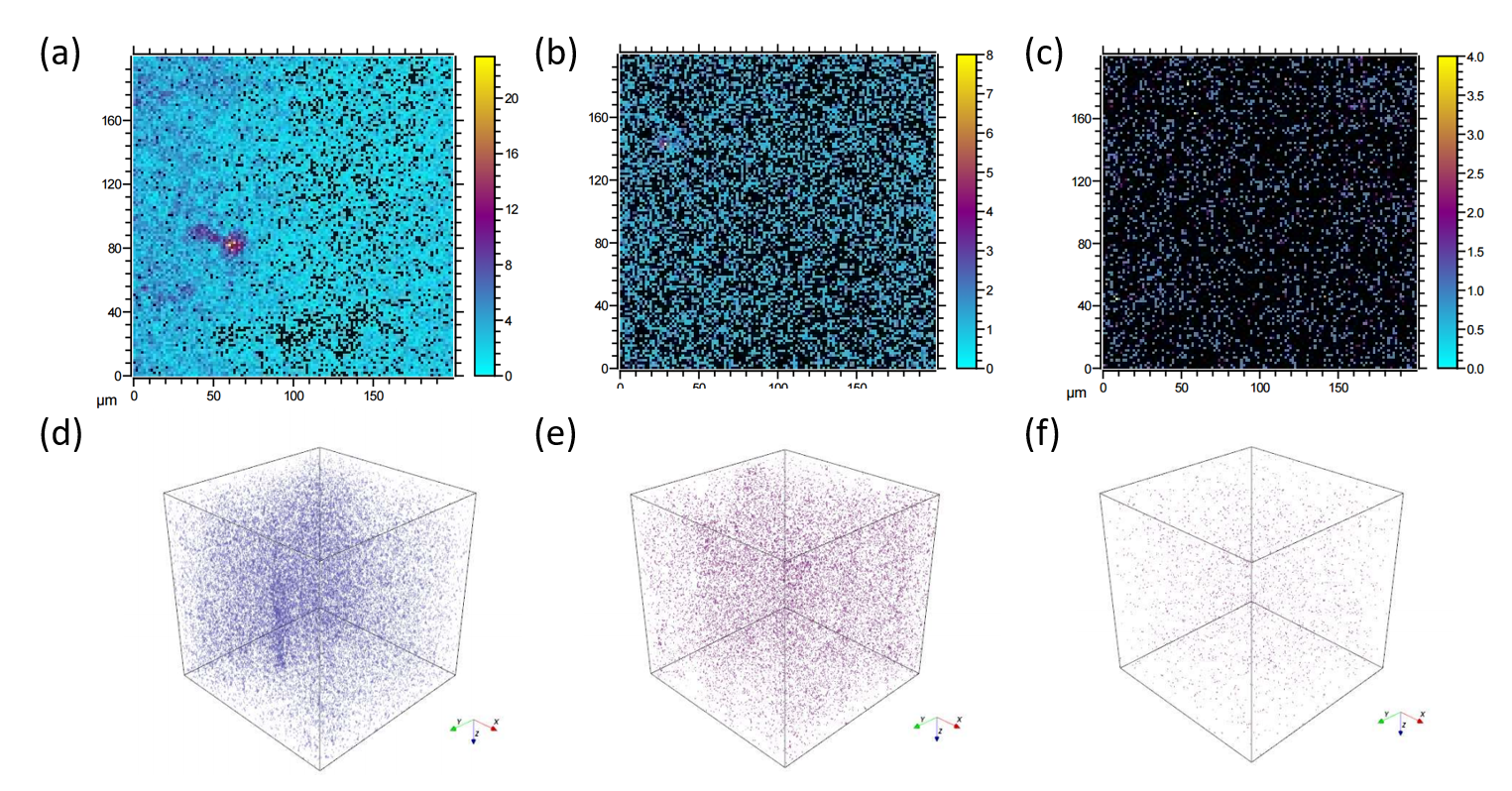


**SI 19.** TGA profiles of PB powders after exposure to an ambient atmosphere (RH 20%) for 1 h


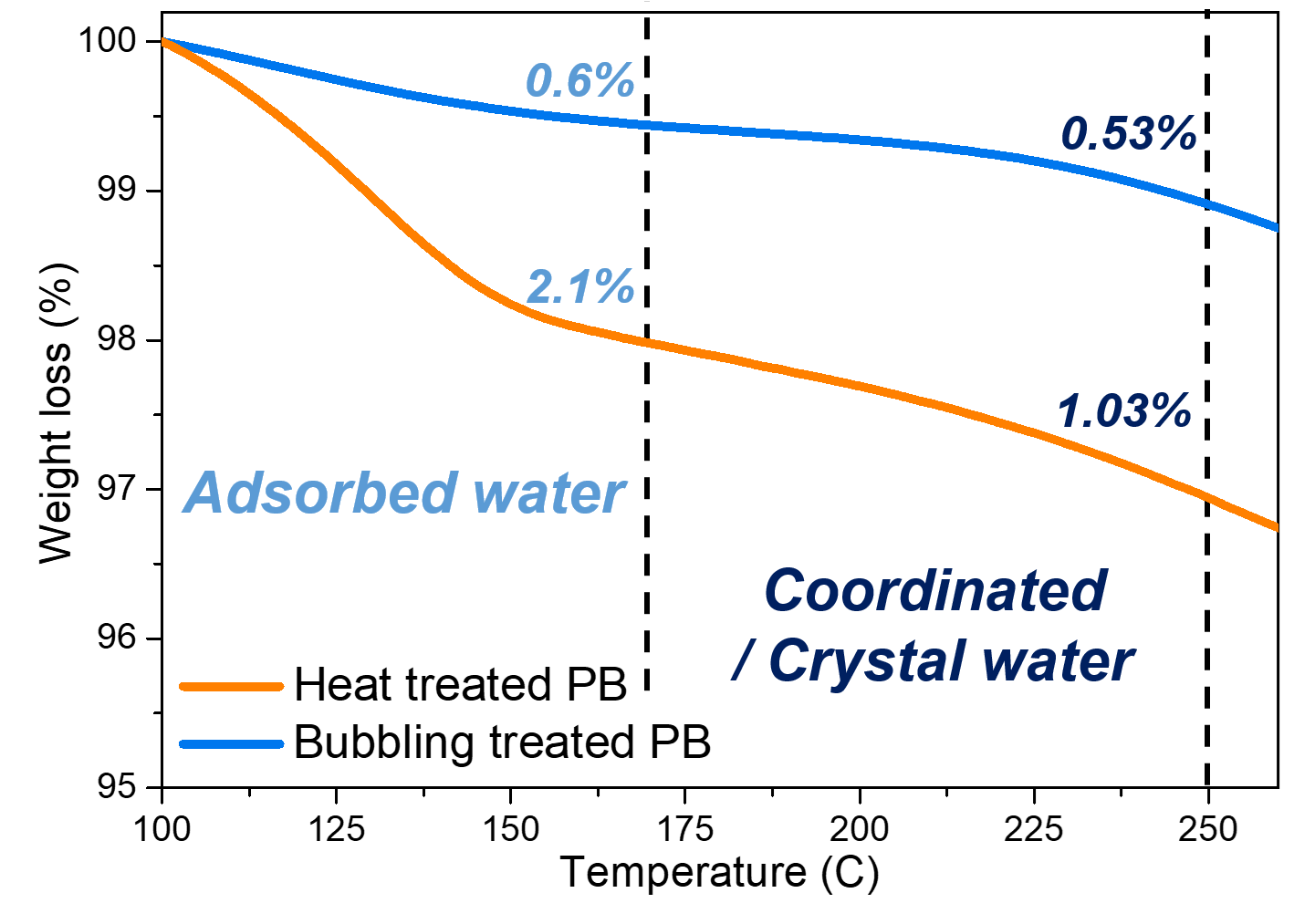


**SI 20.** ICP-OES analysis of heat treated PB and bubbling treated PB


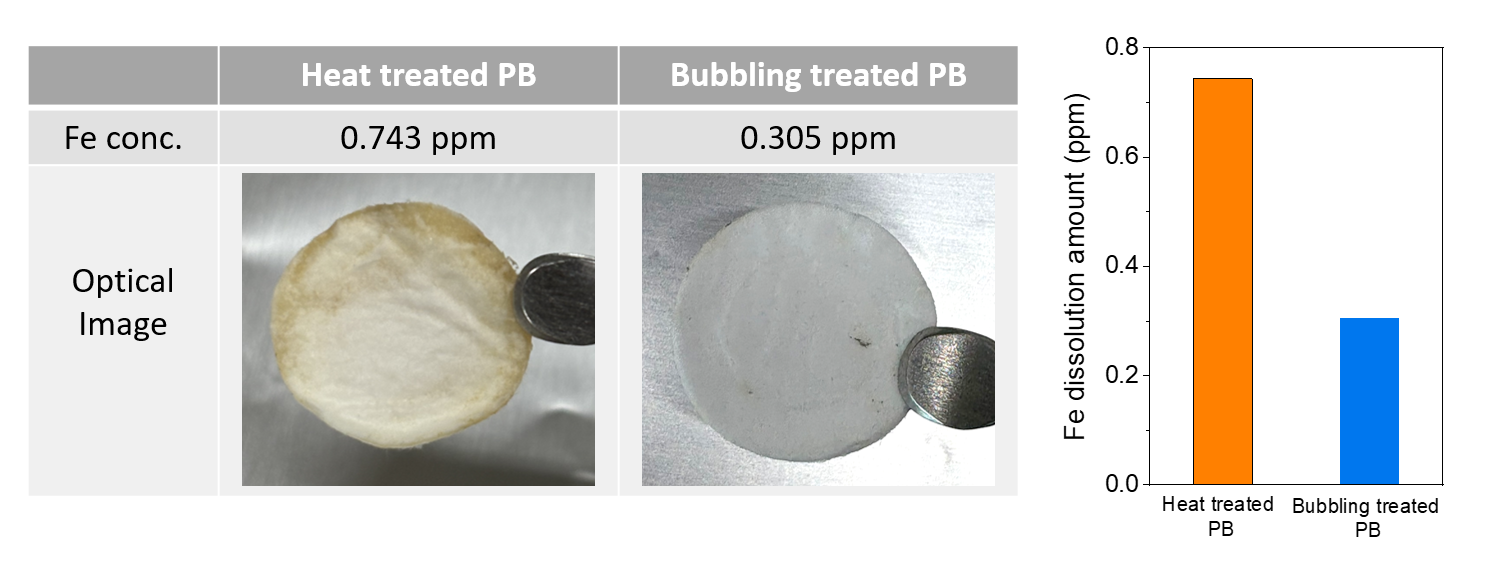


SI 21. **(a)** TGA of untreated and bubbling-treated PB (co-precipitation), **(b)** 1st and 2nd voltage profiles of bubbling-treated PB.


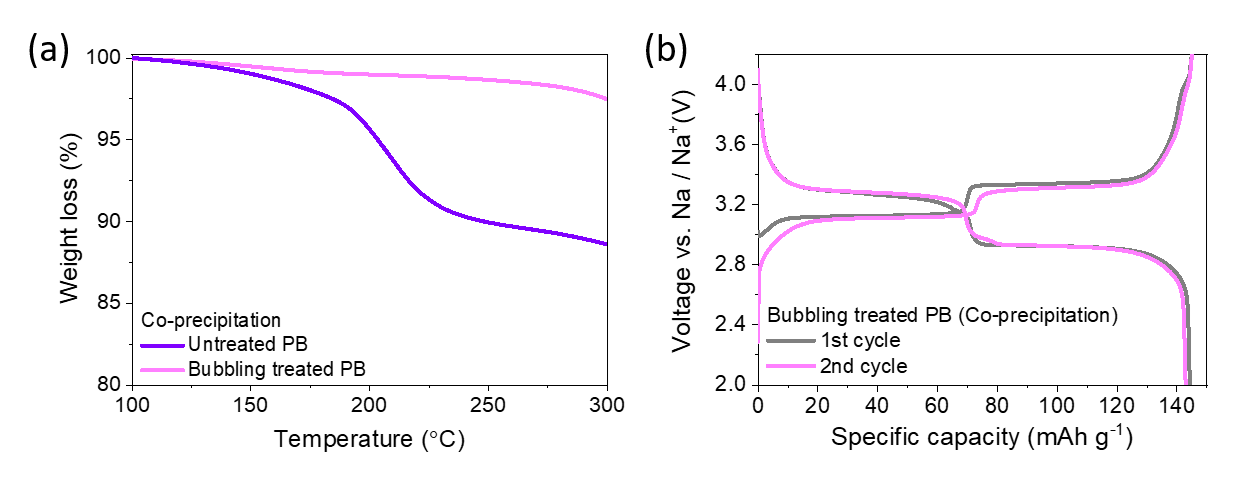


**Table S1**. Summary of reported non-aqueous solvent–based PB synthesis methods

| Method | Solvent | Crystal water content (~260°C) | Crystal structure | Temperature/  Rxn time | Ref | |
| --- | --- | --- | --- | --- | --- | --- |
| Microwave-assisted synthesis | Anhydrous ethanol | 12 wt% | Cubic | 80~120°C/  4 hours | [22] |  |
| Self-assembly | Glycerol / DI water /  Anhydrous ethanol | 15 wt% | Cubic | 140°C/  24 hours | [23] |  |
| Solvothermal synthesis | DI water /  Ethylene glycol | 11 wt% | Rhombohedral | 150°C/  48 hours | [21] |  |
| Solvothermal synthesis | DI water/  Ethylene glycol | 12 wt% | Cubic | 80°C/  24 hours | [24] |  |

**Table S2.** Literature comparison of representative heat-treated PB cathodes and key testing parameters.

| 1 C-rate | Particle size | Crystal water content (~260°C) | Mass loading | Temperature | Ref | |
| --- | --- | --- | --- | --- | --- | --- |
|  | 1~2 μm | 1.32 wt % | 2 mg /cm^2^ | 130°C | [15] |  |
| 100 mA /g | 1~2 μm | 0~1 wt % | 2 mg /cm^2^ | 270°C | [16] |  |
| 120 mA /g | 1~2 μm | 1.6 wt % | 1-2 mg /cm^2^ | 325°C | [18] |  |
| 120 mA /g | 1~2 μm | 10.9 % | 1-2 mg /cm^2^ | 160°C | [25] |  |
| 150 mAh / g | 3~4 μm | 0~1 wt % | 2 mg /cm^2^ | 200°C | [26] |  |
| **140 mAh / g** | **3~4 μm** | **1.4 wt %** | **2-3 mg /cm^2^** | **80°C** | **This work** |  |
